# Fuel-Free Rolosense: Viral Sensing Using Diffusional Particle Tracking

**DOI:** 10.1021/acssensors.5c02311

**Published:** 2025-11-05

**Authors:** Selma Piranej, Krista Jackson, Luona Zhang, Jacob Kæstel-Hansen, Frank Sommerhage, David DeRoo, Nikos S. Hatzakis, Khalid Salaita

**Affiliations:** † Department of Chemistry, 1371 Emory University, Atlanta, Georgia 30322, United States; ‡ Wallace H. Coulter Department of Biomedical Engineering, Georgia Institute of Technology and Emory University, Atlanta, Georgia 30322, United States; § Department of Chemistry, University of Copenhagen, Copenhagen 2100, Denmark; ∥ Center for 4D Cellular Dynamics, University of Copenhagen, Copenhagen 2100, Denmark; ⊥ Novo Nordisk Center for Optimised Oligo Escape and Control of Disease, University of Copenhagen, Copenhagen 2100, Denmark; # Primordia Biosystems, Costa Mesa, California 92626, United States

**Keywords:** aptamers, biosensing, Brownian diffusion, motion-based detection, single-particle tracking, exhaled breath condensate, viral detection

## Abstract

High-sensitivity viral diagnostics typically use PCR to detect and amplify viral nucleic acids which requires fluorescence reporters, enzymatic amplification, specialized equipment and can be time-consuming. In this work, we describe fuel-free (FF) Rolosense, a diagnostic approach that leverages mechanical force sensing as a fundamental transduction mechanism. We use the Brownian motion of aptamer-coated microparticles on an aptamer-modified surface for viral detection. The microparticles function as both the sensing and transduction elements, reporting specific molecular interactions where the presence of viral particles stalls their motion by cross-linking them to the surface. FF-Rolosense harnesses biased motion and thermal fluctuations to achieve rapid, sensitive, and specific detection of intact virions—the active agents of infection. This approach represents a fundamental shift from conventional diagnostic methods and demonstrates a limit of detection as low as 10^3^ copies/mL for SARS-CoV-2 variants, including BA.1 and BA.5, and effectively differentiates SARS-CoV-2 from other viral pathogens such as Influenza A, HCoV OC43, and 229E. We also show that FF-Rolosense readout is amenable to deep learning analysis revealing single particle viral binding events. Finally, we demonstrate potential for point-of-care and home-based applications by using a 3D-printed brightfield microscope, *Roloscope*, for FF-Rolosense readout. Taken together, this work shows a complementary strategy for viral diagnostics that employs a mechanical mechanism of transduction.

## Graphical abstract

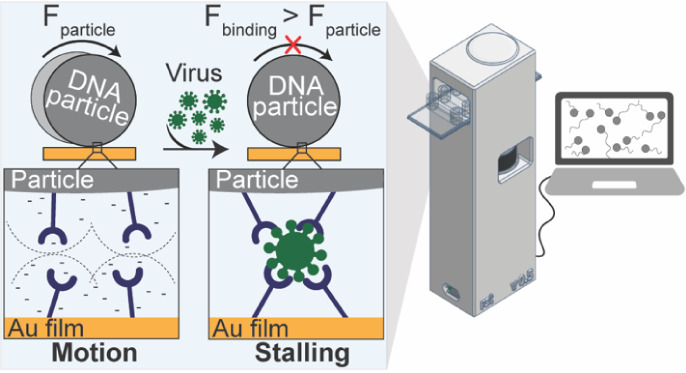

Emerging and reemerging viruses pose persistent and unpredictable threats to global public health, underscoring the urgent need for diagnostic tools that are not only rapid and sensitive but also accessible and amenable to frequent use.^1–3^ Polymerase chain reaction (PCR)^4^ and lateral flow assays (LFAs)^5,6^ remain the dominant technologies in clinical and at-home testing, respectively. While PCR offers excellent sensitivity, it requires thermocycling equipment and skilled operation. LFAs, though more user-friendly and equipment-free, often suffer from limited sensitivity and can struggle to differentiate between closely related viral targets.^7^ Moreover, both approaches typically rely on endpoint measurements—static snapshots of molecular binding events that are chemically or enzymatically fixed, washed, or amplified.^8^ This snapshot paradigm limits the ability to continuously monitor molecular interactions and specifically, low affinity interactions that are highly transient in nature. Accordingly, the current paradigm encounters challenges when detecting low-abundance targets at early stages of infection, when intervention is most effective.

To overcome these limitations, we introduce Fuel-Free (FF) Rolosense, a motion-based viral detection platform that leverages passive Brownian motion to enable real-time, dynamic sensing of viral targets. Building on the the previously developed Rolosense assay^9^ which employed RNase H cleavage to enzymatically propel DNA-functionalized particles across an RNA-coated surface^10–15^ FF-Rolosense eliminates fuel strands, enzymes, and cold-chain storage and instead relies solely on diffusional particle behavior to report on molecular interactions. Microparticles (5 μm silica or polystyrene beads) are functionalized with DNA aptamers targeting specific viruses and introduced onto a chip surface bearing corresponding DNA or aptamer capture sequences. In virus-free samples, particles diffuse freely across the surface. However, when target virus is present, it acts as a multivalent bridge between particle and chip surface, effectively restricting diffusion. The transition from mobile to immobilized (or transiently immobile) states serves as a direct and quantitative indicator of viral presence.

A distinguishing feature of FF-Rolosense is its dynamic sensing mechanism: continuous particle motion enables constant molecular interrogation of the environment, drastically increasing the likelihood of binding even at low target concentrations. This real-time mobility-based approach not only improves sensitivity but also eliminates the need for wash steps or signal amplification, significantly streamlining the assay workflow. Moreover, FF-Rolosense is compatible with exhaled breath condensate (EBC), a noninvasive and highly accessible sample type, making it well-suited for frequent and at-home testing.^16–18^ The use of 5 μm microparticles confers several practical advantages: their sedimentation is easily controlled,^19,20^ their large surface area supports high-density aptamer loading,^21^ and their well-characterized diffusion profiles allow robust motion analysis.^20,22,23^ To quantitatively interpret these motion patterns, we employed DeepSPT,^24^ a deep learning-based single-particle tracking framework, which accurately differentiates mobile from restricted particle trajectories. Our results reveal a clear shift in diffusional behavior upon viral binding, with the proportion of arrested particles scaling with viral concentration. This correlation enables a highly sensitive, motion-based readout without requiring labels or enzymatic amplification.

Here, we show that FF-Rolosense achieves a detection limit of 10^3^ copies/mL for clinically relevant respiratory viruses, including SARS-CoV-2 variants (BA.1, BA.5, XBB.1.5) and respiratory syncytial virus A (RSV A). The platform demonstrates high specificity, distinguishing these targets from nonspecific viruses such as Influenza A and common human coronaviruses OC43 and 229E. Furthermore, FF-Rolosense is modular and programmable as aptamers with high specificity can be easily swapped and modified to enable multiplexed detection. The assay is compatible with a portable, low-cost brightfield imager (*Roloscope*), enabling deployment in resource-limited settings and point-of-care environments. Altogether, FF-Rolosense represents a new class of motion-based diagnostics that are fuel-free, enzyme-free, and real-time designed for accessibility, scalability, and sensitivity. By capturing dynamic molecular interactions through changes in particle mobility, this platform opens new opportunities for noninvasive, high-frequency viral monitoring in both clinical and at-home settings.

## Results and Discussion

### Developing FF-Rolosense Assay

We developed FF-Rolosense as a motion-based viral detection assay by functionalizing both 5 μm silica microparticles and gold-coated substrates with DNA aptamers specific to SARS-CoV-2 spike protein^25^ (Figure 1a–b and Table S1). Silica particles were first modified with azidoacetic acid NHS ester and subsequently conjugated to alkyne-modified DNA aptamers via copper click azide–alkyne cycloaddition (CuAAC). In parallel, gold substrates were functionalized with DNA aptamers and then subsequently passivated with thiolated polyethylene glycol (SH-PEG) to create a high-density, nonfouling DNA-functionalized surface. To establish baseline mobility and optimize buffer conditions, we systematically studied particle motion on DNA-coated chips under various PBS concentrations (0.1×, 0.2×, 0.5×, 1×) (Figure S1). While particles in low-ionic strength conditions (0.1× to 0.5× PBS) displayed relatively high lateral mobility, increased ionic strength led to progressively reduced net displacement, with particles in 1× PBS showing clear signs of partial adhesion and significantly dampened motion. This trend reflects the role of electrostatic screening: as salt concentration increases, repulsion between the negatively charged DNA layers on the particle and surface is inhibited, promoting nonspecific binding. Additionally, when we tested silica particles that lacked any DNA aptamer coating, we observed immediate and near-complete sticking on the DNA-modified chip (Figure S2), highlighting that surface DNA functionalization is essential to maintaining motion by preventing nonspecific adhesion. These results align with prior reports showing that high-density DNA coatings can produce electrostatic repulsion strong enough to vertically separate particles from surfaces by hundreds of nanometers,^26^ which likely helps maintain lateral diffusivity in the FF-Rolosense assay.

**Figure 1:**
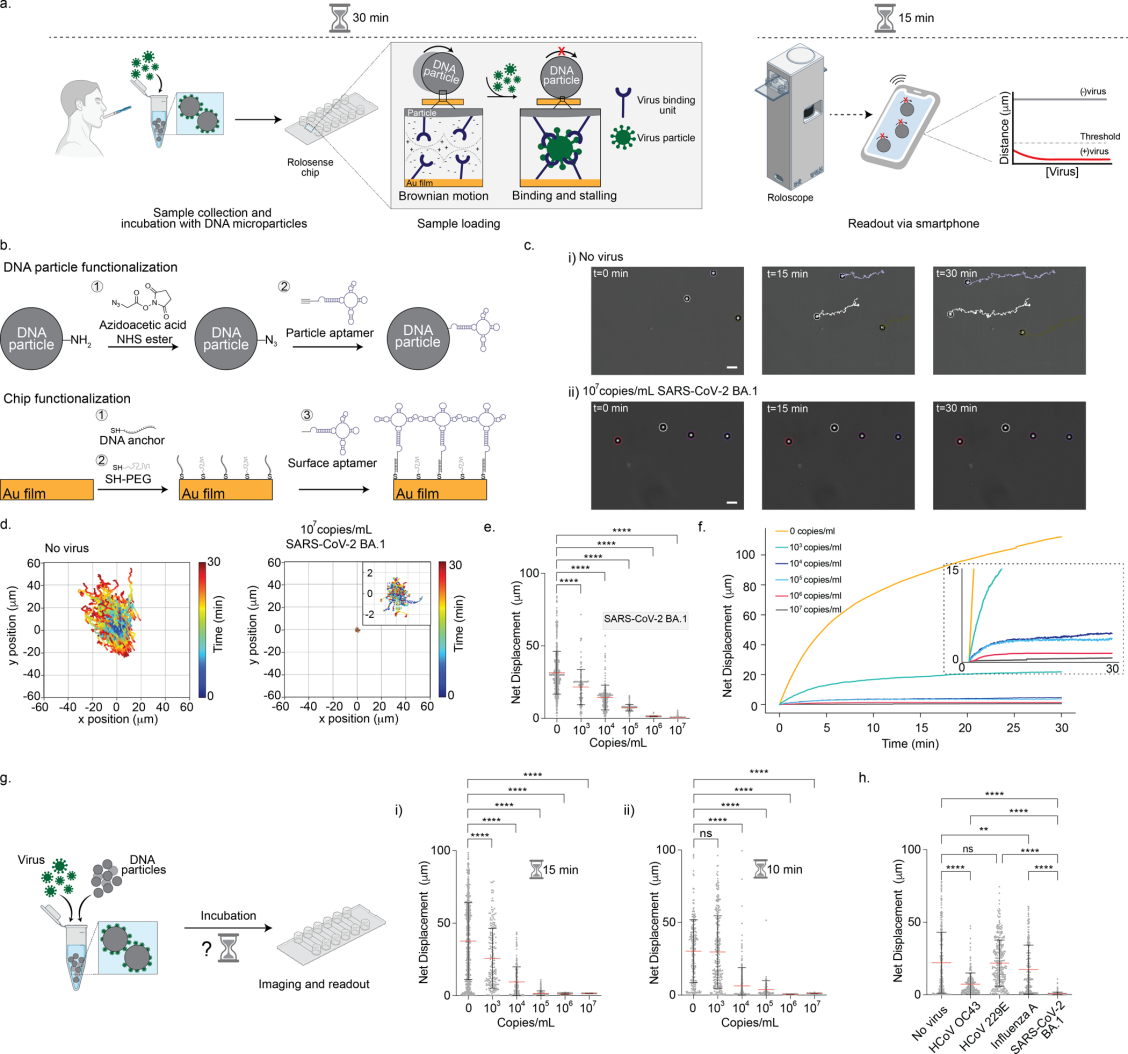
DNA particles acting as chemical signal transducers. (a) Overview of the FF-Rolosense workflow. DNA-functionalized 5 μm silica microparticles are incubated with patient-derived samples (e.g., exhaled breath condensate) for 30 min at room temperature. In the absence of virus, particles exhibit biased Brownian motion; in the presence of virus, binding events cause mechanical stalling and reduced displacement. Particle motion is recorded using brightfield time-lapse imaging, and diagnostic readout is derived from quantifying displacement. (b) Surface chemistry schematic showing the functionalization of DNA particles with aptamers via NHS-azide and CuAAC chemistry (top), and the chip surface using thiol-gold interactions and DNA hybridization (bottom). (c) Representative brightfield images and trajectory overlays for virus-free samples (top row) and 10^7^ copies/mL SARS-CoV-2 BA.1 (bottom row), captured at 0, 15, and 30 min. Scale bar is 10 μm. (d) Plots showing the trajectories of particles with no virus and 10^7^ copies/mL of UV-inactivated SARS-CoV-2 BA.1 strain spiked in exhaled breath condensate. All the trajectories are aligned to the 0,0 (center) of the plots for time = 0 min. Color indicates time (0 → 30 min). (e) Plots of net displacement of over 300 particles incubated with ranging concentrations of SARS-CoV-2 BA.1. UV-inactivated SARS-CoV-2 samples were spiked in exhaled breath condensate and incubated with the particles at room temperature for 30 min. Each sample was performed in triplicate. **** indicates *p* < 0.0001. (f) Displacement curves as a function of time for different virus concentrations incubated with particles (*N* = 100) from a single trial, showing progressive reduction in motion with increasing viral concentration. Inset highlights the 0–15 min region where differences begin to emerge. (g) Schematic of sample generation which includes incubation of virus with DNA microparticles ranging at 10 and 15 min. (i) Plots of net displacement of ∼300 motors with different concentrations of UV-inactivated SARS-CoV-2 BA.1 in exhaled breath condensate at 10 min incubation time and (ii) at 15 min incubation time. ns and **** indicate not statistically significant and *p* < 0.0001, respectively. Experiments were performed in triplicate. (h) Plot showing the net displacement for over 100 particles incubated with 10^5^ copies/mL of UV-inactivated HCoV OC43, HCoV 229E, influenza A, and SARS-CoV-2 BA.1 spiked in exhaled breath condensate. All measurements were performed in triplicate. ns, **, and **** indicate not statistically significant, *p* = 0.0015, and *p* < 0.0001, respectively.

Having established the motion-enabling role of electrostatic repulsion in buffered systems, we next transitioned to exhaled breath condensate (EBC) as the primary sample matrix for all subsequent virus detection experiments. EBC was collected noninvasively using the commercially available RTube device (Respiratory Research, Austin, TX, USA), which condenses breath aerosols through passive cooling. This sample type has inherently low ionic strength and requires no active patient participation beyond normal breathing, making it an ideal biological fluid for real-world diagnostics.^27,28^ In control experiments using EBC without virus, we observed high particle mobility and long trajectories, with net displacements remaining stable for up to 4 h of incubation (Figure S3). This indicates that the particles remain functionally active and nonaggregated over time, and that the assay can operate unperturbed in EBC at room temperature. While analyzing baseline particle trajectories in EBC, we initially observed a consistent directional drift even in virus-free conditions (Figure 1c,d). Further investigation revealed this to be an artifact of subtle microscope stage tilt. As shown in Figure S4, even minor angles (0.3°–0.7°) introduced gravitational bias, leading to directional displacement unrelated to molecular interactions. We then incubated DNA aptamer-coated particles with 10^7^ copies/mL of UV-inactivated SARS-CoV-2 BA.1 and monitored their motion on the chip. Compared to the long trajectories in the no-virus control, particles exposed to virus exhibited confined motion and significantly lower net displacements (Figure 1c,d). In some cases, particles remained localized over the full 30 min time-lapse period, consistent with multivalent bridging between surface and particle-bound aptamers in the presence of target virus.

We then evaluated the assay’s detection sensitivity by testing a dilution series of SARS-CoV-2 BA.1 (10^3^–10^7^ copies/mL) in EBC with a 30 min incubation (Figure 1e,f). No dedicated sedimentation wait is required for 5 μm silica particles; they settle to the chip surface in under a minute. Accordingly, we begin imaging immediately after adding the microparticles to the chip, with early frames capturing the tail end of settling (Figure S5). Figure 1e shows compiled single-particle tracking results from three independent experimental trials, with each point representing the net displacement of a single DNA particle. The displacement data was extracted from brightfield time-lapse recordings, using a custom image analysis pipeline described in Figure S6. A clear trend emerges across the dilution series: as viral concentration increases, the average net displacement of particles decreases significantly. At the highest concentration (10^7^ copies/mL), nearly all particles are confined, with net displacements below 10 μm, whereas in the virus-free control, many particles travel well beyond 50 μm. Notably, the 10^3^ copies/mL group already shows statistically significant reduction (****p < 0.0001) in motion compared to control, indicating that FF-Rolosense is sensitive enough to detect virus at this lower limit of detection. To better understand how particle mobility evolves over time, we plotted the average net displacement as a function of time from one representative trial (Figure 1f). In the absence of virus, particles show steady increases in displacement over the 30 min period, reflecting continuous biased Brownian motion. In contrast, the virus-exposed samples exhibit an initial rise in net displacement over time followed by plateauing, with higher viral concentrations flattening out at earlier time points.

Next, we aimed to test the effect of incubation time on viral binding, specifically testing 15 and 10 min incubation periods (Figure 1g). We found that 15 min incubation preserved sensitivity, with net displacement remaining significantly reduced at 10^3^ copies/mL compared to controls. However, at 10 min, the difference between the no-virus control and low viral concentration was no longer significant, indicating that shorter incubation times limit the probability of particle-virus binding events. Finally, to assess specificity, we challenged the assay with other respiratory viruses, including HCoV OC43, HCoV 229E, and Influenza A (Figure 1h). Only SARS-CoV-2 variants (BA.1) led to measurable stalling of particle motion, while nontarget viruses had little to no effect (Figure 1h). This confirms the high selectivity of the DNA aptamers and suggests that FF-Rolosense is not impacted significantly by nonspecific viral interactions or general viral load.

### Using FF-Rolosense to Detect RSV A Virus

Further demonstrating the programmability and target specificity of FF-Rolosense, we adapted the assay to detect respiratory syncytial virus A (RSV A). By replacing the aptamer sequences on both the 5 μm silica microparticles and the gold chip surface with ones targeting RSV A, we reconfigured the system for a new viral target (Figure 2a). The aptamer used for this study was previously reported to bind RSV A with high affinity^29^ and its predicted secondary structure, rendered using NUPACK, is shown in Figure 2b. This simple modification underscores FF-Rolosense’s versatility allowing viral retargeting through a “plug-and-play” aptamer swap without altering any other component of the assay.

**Figure 2:**
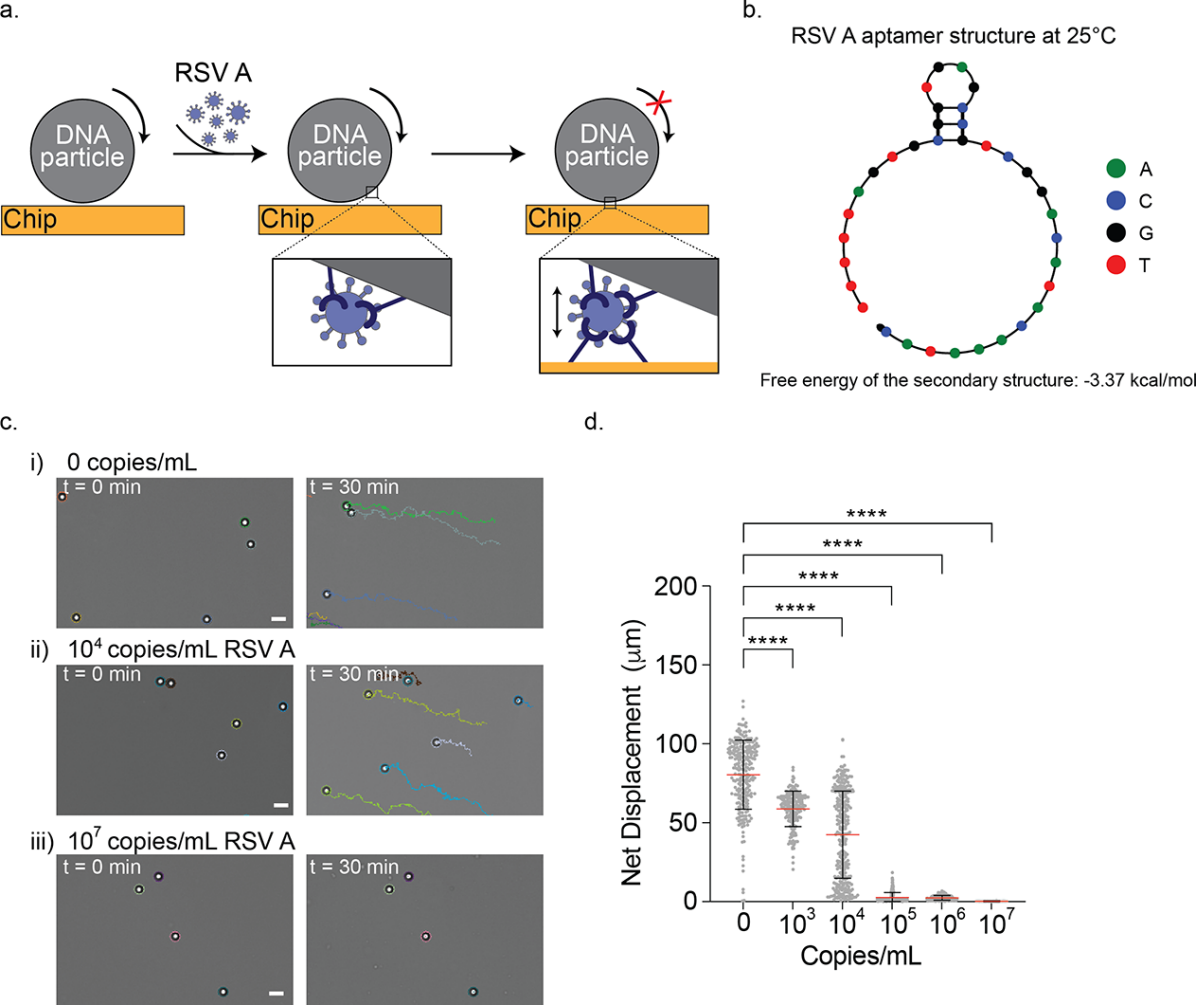
Detection of RSV A viruses. (a) Schematic illustration of the FF-Rolosense detection mechanism for RSV A. DNA particles and chip surfaces are functionalized with an RSV A–specific aptamer. In the presence of virus, multivalent interactions between viral particles and aptamers on both surfaces lead to mechanical stalling of the DNA microparticles. (b) Predicted secondary structure of the RSV A aptamer at 25 °C in 1X PBS (146 mM Na^+^ and 4.5 mM K^+^) showing a stable fold with a calculated free energy of −3.37 kcal/mol. (c) Representative brightfield images and particle trajectories at 0 and 30 min of incubation with varying concentrations of UV-inactivated RSV A in exhaled breath condensate: (i) 0 copies/mL, (ii) 10^4^ copies/mL, and (iii) 10^7^ copies/mL. Particles exposed to higher virus concentrations show visibly reduced motion. Scale bar is 10 μm. (d) Plots showing the net displacement for over 300 particles incubated with different concentrations of UV-inactivated RSV A spiked in exhaled breath condensate. The error bars and the red lines represent the standard deviation and the mean of the distribution, respectively. ns and **** indicate not statistically significant and *p* < 0.0001, respectively.

To assess RSV A detection, we incubated the modified particles with varying concentrations of virus in EBC and tracked their motion on the aptamer-functionalized surface. Representative brightfield images and particle trajectories are shown in Figure 2c for viral concentrations of 0, 10^4^, and 10^7^ copies/mL at both the start (*t* = 0 min) and end (*t* = 30 min) of imaging. In the absence of virus, particles exhibit long, free-diffusing trajectories, consistent with unrestricted biased Brownian motion. As viral concentration increases, the particle paths become progressively shorter and more confined, indicating increasing degrees of binding-mediated motion inhibition. At 10^7^ copies/mL, particles appear nearly stationary. These qualitative results are supported by the quantitative displacement data shown in Figure 2d, which compiles net displacements for individual particles across three trials. A clear inverse trend is observed: higher concentrations of RSV A produce lower mean particle displacements. Significant motion inhibition is observed starting at 10^3^ copies/mL, confirming that FF-Rolosense retains its sensitivity when repurposed for a different viral target. We also functionalized 6 μm polystyrene particles with aptamers targeting influenza A^30^ and conducted detection experiments in EBC (Figure S7). Despite the change in particle material, size, and optical properties, the assay remained functional, successfully detecting influenza A at concentrations of 10^4^ copies/mL. This result demonstrates multiplexing capabilities of FF-Rolosense as it is not limited to a specific particle type and can be extended to a range of commercially available bead formats.

### Optimizing Sensitivity of FF-Rolosense Assay by Changing Aptamer Span Length

To explore parameters that could improve the sensitivity of FF-Rolosense, we investigated the effect of aptamer span length by systematically varying the linker region between the particle and the aptamer binding domain. Specifically, we compared two polyT spacers—T_20_ and T_40_—and a PEG_36_ spacer of comparable contour length (∼12 nm), each incorporated into both the particle- and surface-bound aptamers (Figures 3a and S8). We hypothesized that altering the spacer length and chemistry would influence both the baseline diffusivity of DNA particles and their ability to engage in multivalent binding with the target virus.

**Figure 3:**
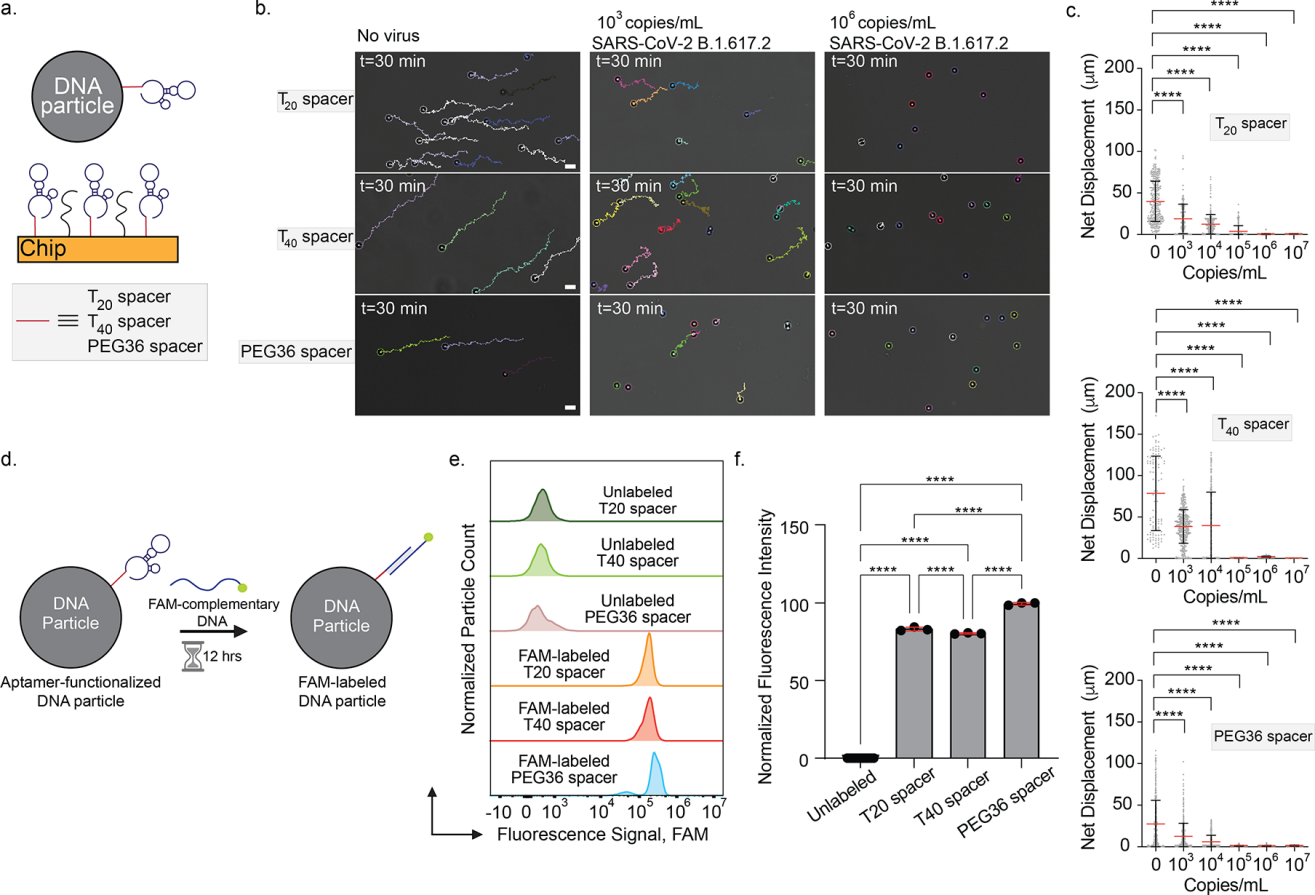
Effect of DNA aptamer span on SARS-CoV-2 detection. (a) Schematic of DNA microparticles functionalized with aptamer that has T_20_, T_40_ or PEG_36_ spacer. The chip surface is functionalized with the same aptamer and passivated with thiolated PEG. (b) Representative brightfield images and particle trajectories after 30 min of incubation with 0, 10^3^, and 10^6^ copies/mL of UV-inactivated SARS-CoV-2 B.1.617.2 (Delta variant) in exhaled breath condensate. Each row corresponds to a different spacer condition. (c) Plots of net displacement of ∼300 DNA particles per condition, showing spacer-dependent motion inhibition as a function of viral load. Scale bar is 10 μm. Red lines and error bars represent the mean and standard deviation, respectively. **** indicates *p* < 0.0001. (d) Schematic of the fluorescence hybridization assay used to quantify aptamer surface density on DNA particles. FAM-labeled complementary DNA was hybridized overnight to particles functionalized with each spacer type. (e) Flow cytometry histograms showing fluorescence signal distribution across FAM-labeled particles with T_20_, T_40_ or PEG_36_ aptamers, compared to unlabeled controls. (f) Bar plot of normalized fluorescence intensity across spacer types, indicating significantly higher aptamer density on PEG_36_-modified particles. Each bar represents the mean of three replicates; **** indicates *p* < 0.0001.

To test this, we functionalized DNA particles and chip surfaces with aptamers containing each spacer type and incubated them with SARS-CoV-2 B.1.617.2 (Delta variant) at various concentrations for 30 min in EBC. Note that for these optimization experiments the particle and the chip were functionalized with an aptamer^31^ that has high affinity for the Delta variant. In the absence of virus, particles with the longer T_40_ spacer exhibited the highest net displacement over 30 min, followed by T_20_ and PEG_36_-modified particles (Figure 3b, left panels). This increase in baseline diffusivity likely results from the extended interaction radius afforded by longer spacers, which reduces steric hindrance and elevates particle-surface separation. All three spacer configurations enabled sensitive detection of SARS-CoV-2, with net displacement decreasing significantly as viral concentration increased (Figure 3b–c). However, particles functionalized with PEG_36_ spacers showed a more pronounced reduction in displacement across the viral titration series, suggesting enhanced sensitivity. At 10^3^ copies/mL, PEG_36_-coated particles already exhibited marked stalling, while T_20_ and T_40_ required slightly higher concentrations for comparable inhibition. This trend was confirmed across replicate data sets, supporting the conclusion that PEG_36_ linkers enhance viral responsiveness (Figure 3c).

To better understand this enhanced performance, we quantified aptamer loading on particles using a hybridization-based fluorescence assay. DNA particles functionalized with each spacer variant were hybridized to FAM-labeled complementary strands overnight (Figure 3d), and single-particle fluorescence intensities were measured (Figure 3e–f). Notably, PEG_36_-aptamer particles displayed significantly greater fluorescence signals compared to both T_20_ and T_40_ particles, indicating a higher aptamer surface density. This result supports findings that flexible, hydrophilic PEG spacers optimize aptamer packing by minimizing interstrand electrostatic repulsion and steric crowding, particularly at curved surfaces.^21,32^ We benchmarked particle fluorescence to a previously quantified DNA-motor reference.^10^ Our results show that the PEG36-aptamer particles are ∼30–35% of that reference loading (Figure S9), which given the established density of ∼7 × 10^6^ strands per 5-μm bead for the reference, it corresponds by scaling to ∼2–2.5 × 10^6^ strands per aptamer particle. We also tested whether this higher particle-based aptamer density was also observed at the surface level, we compared the surface fluorescence intensity of PEG_36_-functionalized chips to those coated with the no-spacer aptamer using the same DNA sequence (Figure S10). Surprisingly, both configurations exhibited similar levels of surface signal, suggesting that the PEG spacer does not adversely affect aptamer immobilization on flat surfaces. Increased aptamer density directly contributes to enhanced polyvalency—the ability of each particle to simultaneously engage multiple viral spike proteins. Higher polyvalency increases the likelihood of binding events, even at low viral loads, leading to more robust motion inhibition.

To directly quantify how PEGylation affects binding affinity, we performed a solution-phase binding assay using GFP-expressing SARS-CoV-2 pseudoviruses and flow cytometry (Figure S11). As shown in Figure S11b, particles coated with non-PEGylated aptamers yielded an apparent dissociation constant of 10 fM, while PEG_36_-modified aptamers improved affinity even further, achieving a *K*_d,app_ of 7 fM. Scrambled controls showed minimal binding across all concentrations. Median fluorescence measurements at 0.03 pM virus concentration confirmed specific and high-affinity recognition by PEGylated aptamers compared to all controls (Figure S11c). These results were consistent with the independent replicate shown in Figure S12, which validates the enhanced binding of PEG-modified aptamers to BA.1 viral targets. Furthermore, the PEG_36_-modified aptamer was able to detect distinct Omicron subvariants, including BA.5.1 and XBB.1.5 (Figure S13). Taken together, these data confirm that PEGylation not only increases aptamer loading but also enhances molecular binding affinity, likely by improving the aptamer’s structural flexibility and spatial accessibility.

Because the assay operates in EBC, we next examined affinity across matrices noting that aptamer folding and thus apparent affinity depends on ionic strength. Using the same solution-phase binding assay, we fit the background-subtracted data to a one-site Hill model with slope fixed to 1 (Figure S14). In 1× and 0.1× PBS, titrations cleanly plateau and yield well-defined *K*_d_ values of 11 fM and 17 fM, respectively. In EBC, the curve is right-shifted and reaches a shallower plateau. The maximal binding signal is ∼60% lower than in 1X PBS, indicating reduced capacity. The fitted *K*_d_ (73 fM) is consistent with weaker apparent affinity, but it has wider uncertainty because the low-concentration region is noisier. Even so, FF-Rolosense maintains a detection limit of 10^3^ copies/mL in EBC because readout is governed by multivalent interactions in which aptamer-dense beads and chip present many binding sites per virion, so even rare encounters form multibond attachments that produce long stalls.

### Deep Learning-Based Analysis of Fuel-Free Rolosense Diffusion in the Presence of Viral Particles

To analyze how SARS-CoV-2 BA.1 modulates the diffusional behavior of DNA particles in the FF-Rolosense assay, we applied DeepSPT,^33^ a deep learning-based single-particle tracking framework designed to extract and classify motion states from particle trajectories (Figure 4a). Notably, this approach enables frame-level segmentation of motion, allowing precise identification of the timing of transitions such as virus encounters. These experiments were performed using DNA particles functionalized with the PEG_36_-aptamer, as this configuration was previously shown to offer improved aptamer density and binding affinity. DeepSPT first segments raw trajectories into temporally distinct motion states, classifying subsegments of trajectories as “free” or “restricted.” It combines the temporal segmentation with a 40-feature diffusional fingerprint to inform a task-specific classifier in mapping diffusional patterns and infer whether a particle has likely encountered a viral target. Representative single-particle trajectory illustrates this classification process (Figure 4b). Freely diffusing particles (blue) explore extended regions of the surface, while restricted particles (red) remain confined to nanometer domains. The transition between free and restricted motion is clear in the trajectory shown, with the corresponding confidence stacked bar plot below indicating a dynamic switch in motion state. A persistently restricted trajectory is confined within a ∼500 nm region, consistent with strong particle-virus binding or localized entrapment. In virus-exposed samples, particles frequently transition from free to restricted motion, as shown in the confidence plot beneath the trajectory. Some particles exhibit persistent restriction, remaining confined for the duration of the time-lapse—likely the result of multivalent binding between the particle and one or more virus particles. Quantitative analysis across viral concentrations revealed a strong correlation between SARS-CoV-2 BA.1 concentration and the fraction of particles classified as persistently restricted (Figure 4c). Interestingly, while the overall virus-associated fraction increases with viral load, the fraction of “persistently restricted” tracks peaks at intermediate concentrations and decreases at the highest concentrations. This suggests that at high viral loads (10^6^–10^7^ copies/mL), many particles are already trapped very early on and therefore do not exhibit transitions captured by DeepSPT’s restricted state detection. In other words, these particles are so strongly or immediately bound that they appear static throughout the time-lapse, bypassing dynamic classification.

**Figure 4:**
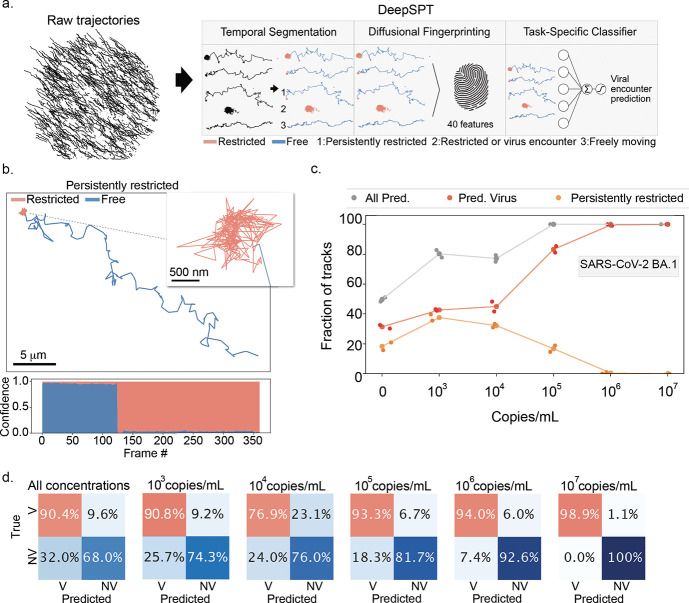
Temporal and diffusional fingerprinting of DNA motor-virus encounters through deep learning. (a) Overview of the DeepSPT framework applied to FF-Rolosense. Raw particle trajectories are analyzed via three modules: (1) temporal segmentation, which annotates particle motion as “Free” (blue) or “Restricted” (red) at each time point; (2) diffusional fingerprinting, which extracts 40 features from segmented tracks; and (3) a task-specific classifier trained to distinguish between virus-exposed and unexposed trajectories based on these features. (b) Representative trajectory of a DNA particle classified as “persistently restricted.” Blue segments represent periods of unrestricted diffusion, while red segments denote virus-induced confinement. Inset shows a zoomed-in region of the restricted phase. The bottom bar displays DeepSPT’s per-frame classification confidence, with the length of red indicating the duration of restricted behavior. (c) Quantification of predicted track behavior across SARS-CoV-2 BA.1 concentrations (0–10^7^ copies/mL). Data are pooled from *n* = 3 biological replicates per condition, with ∼100–400 DNA particles tracked per concentration. Classifications were made from time-lapse brightfield videos (30 min, 0.2 fps). The fraction of tracks classified as virus-associated (red) increases with viral concentration. Meanwhile, the fraction of “persistently restricted” tracks (orange) decreases at the highest concentrations, likely because particles are fully trapped and thus do not exhibit a transition from free to restricted motion. The classifier was trained on virus-free (0 copies/mL) and 10^6^ copies/mL SARS-CoV-2 BA.1 trajectories. (d) Predicted “virus” or “no virus” accuracies at each virus concentration based on temporal and diffusional features. Each matrix represents performance at different viral concentration thresholds. At 10^5^ copies/mL, the classifier achieves >93% accuracy. Prediction was performed using 10-fold cross-validation with random shuffling, and class imbalance was corrected by oversampling.

To evaluate whether these motion phenotypes can classify viral exposure, we trained the task-specific classifier of DeepSPT features to distinguish virus-exposed (V) from nonvirus (NV) trajectories. As shown in Figure 4d, classifier accuracy and true positive rate improved with increasing viral concentration. At concentrations of 10^5^ copies/mL, over 93% of virus-exposed tracks were correctly predicted, while 10^7^ copies/mL yielded near-perfect classification (98.9% true positive rate). Even at 10^3^ copies/mL, performance remained well above baseline substantially exceeding the ∼50% accuracy expected from a random classifier thus demonstrating the system’s sensitivity in detecting early viral interactions. Together, these results demonstrate that DeepSPT can resolve subtle changes in particle mobility induced by viral binding, enabling precise and automated quantification of virus-particle encounters. Importantly, this method captures the continuous and dynamic nature of FF-Rolosense, offering a high-resolution view into how molecular interactions manifest in mesoscale particle behavior.

### Using 3D-Printed Brightfield Imager to Readout FF-Rolosense Performance

As part of our effort to streamline and eliminate reliance on conventional laboratory infrastructure, we developed a portable, low-cost brightfield imager, termed the “*Roloscope*” (Figure 5a). *Roloscope* is a 3D-printed, Wi-Fi-enabled device designed for rapid particle tracking and automated motion analysis (Figure S15). It does not require fluorescence optics, photomultiplier tubes, or high-end objectives. Instead, it uses low-cost brightfield illumination, a 10× microscope lens, and a 2 MP ESP32-CAM-MB module capable of wireless data transmission and onboard image processing. The full assembly supports focus adjustment via RMS-threaded optics, a six-channel chip for multiplexed testing, and USB/SD card connectivity for easy data management (Figure S15). The total footprint of the device is compact (47 mm × 35 mm × 140 mm), making it ideal for point-of-care and field-based deployment.

**Figure 5:**
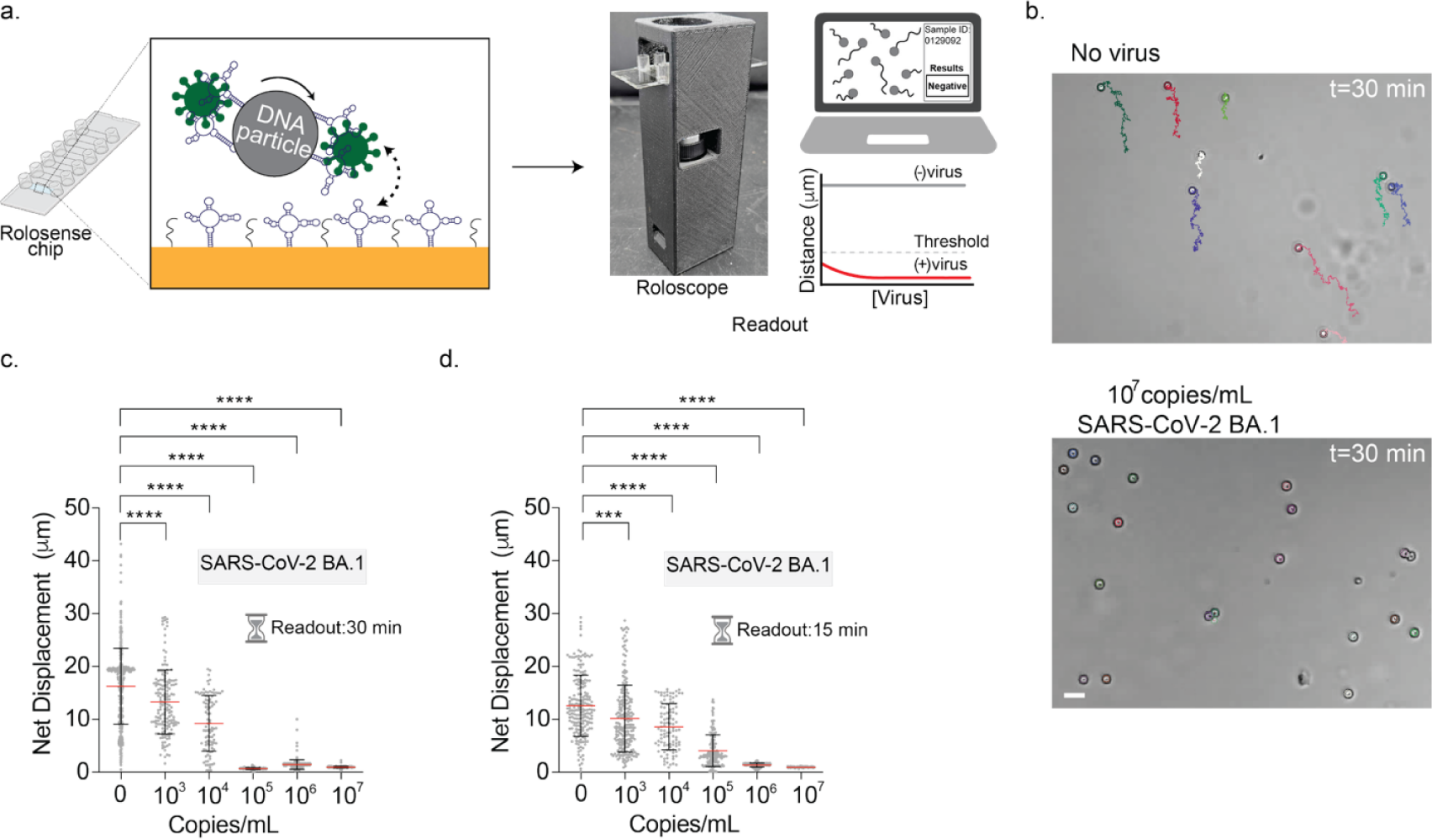
Detecting SARS-CoV-2 BA.1 using a 3D printed brightfield imager. (a) Schematic of the FF-Rolosense assay coupled with the 3D-printed *Roloscope* (photograph). *Roloscope* is a compact, Wi-Fi-enabled brightfield imaging system that captures particle motion and displays results on a connected device. (b) Representative brightfield images with trajectory overlays for virus-free (top) and 10^7^ copies/mL SARS-CoV-2 BA.1 (bottom) samples after 30 min of incubation. Trajectories were randomly assigned unique colors for ease of visualization. Scale bar is 10 μm. (c) Plots showing net displacement for over 100 DNA particles per condition after 30 min of incubation in EBC, followed by a 30 min brightfield time-lapse readout. The error bars and the red lines represent the standard deviation and the mean of the distribution, respectively. **** indicates *p* < 0.0001. (d) Plots of net displacement of >100 particles per condition after 30 min of incubation, followed by a 15 min brightfield time-lapse readout. Red lines represent the mean, and error bars indicate standard deviation. *** and **** indicate *p* < 0.001 and *p* < 0.0001, respectively.

We evaluated the performance of FF-Rolosense using *Roloscope* by incubating PEG_36_-functionalized DNA particles with SARS-CoV-2 BA.1 in exhaled breath condensate across a range of concentrations. After 30 min of incubation, *Roloscope* successfully resolved the mobility differences between virus-negative and virus-positive samples (Figure 5b). Even at 10^3^ copies/mL, particles exhibited markedly restricted motion relative to the no-virus control (Figure 5c). Importantly, the characteristic shift in diffusional behavior remained visible despite the simplified optics, demonstrating the robustness of the signal and the compatibility of FF-Rolosense with low-cost imaging. We next tested whether we could reduce the post-incubation imaging window from 30 to 15 min without compromising assay sensitivity. As shown in the net displacement plots (Figure 5d), particle mobility decreased with increasing viral load. At 10^5^–10^7^ copies/mL, most particles exhibited near-complete stalling within the 15 min window, enabling a full diagnostic result in under 45 min from sample collection to output.

## Conclusion

Conventional viral detection methods often rely on static, enzyme-based, or fluorescence-dependent readouts that introduce complexity and delay. In contrast, FF-Rolosense enables real-time and enzyme-free viral detection without fluorescent reporters by leveraging Brownian motion and virus-induced mechanical stalling of DNA-aptamer-coated microparticles. This dynamic sensing mechanism captures binding events through changes in particle mobility, providing a direct and intuitive readout without the need for amplification or signal development steps.

The assay detects intact viral particles, the infectious units themselves, at concentrations as low as 10^3^ copies/mL, as demonstrated with multiple SARS-CoV-2 variants. It also shows adaptability to other viruses such as Influenza A and RSV A. Importantly, the micron-scale size of the particles constrains them to quasi-2D diffusion at the surface, enhancing the kinetics of virus-particle encounters and improving detection efficiency. To further quantify viral effects on particle motion, we employed DeepSPT, a machine learning-based tracking framework. DeepSPT revealed a strong correlation between viral load and the fraction of persistently restricted trajectories, and reliably distinguished virus-exposed from nonexposed particles. These findings confirm that virus-induced mobility changes provide a robust and quantifiable detection signature.

The FF-Rolosense platform offers practical advantages: real-time signal generation, compatibility with noninvasive samples like exhaled breath condensate, and straightforward imaging via the portable 3D-printed Roloscope device. Note that EBC can be collected comfortably and rapidly using commercial condensers, yielding 1–2 mL of sample per subject after just 10 min,^34^ which is more than sufficient for downstream analysis. By eliminating the need for fluorescence optics, motorized stages, or benchtop instrumentation, Roloscope enables decentralized, on-demand testing with plug-and-play simplicity. When paired with real-time image analysis software, this configuration supports rapid public health screening, field deployment, or even home-based monitoring. As a practical note, the current *Roloscope* does not perform automatic leveling and our tilt characterization shows that residual tilt produces only global field-wide drift not the confined/immobilized states that constitute the positive detection signature.

Despite its advantages, FF-Rolosense also faces practical limitations. First, the assay relies on the statistical analysis of hundreds to thousands of microparticles due to inherent heterogeneity in particle fabrication and functionalization. Second, aptamer specificity can be affected by viral evolution, particularly in surface-exposed antigens prone to mutational drift.^35,36^ Third, while aptamers are relatively straightforward to generate through SELEX,^37^ not all viruses have high-affinity aptamers currently available, which may limit near-term applicability to emerging pathogens. Environmental factors may also affect performance. For instance, ionic strength of the sample buffer influences electrostatic repulsion between particles and the chip, and high-salt conditions could promote nonspecific sticking, complicating real-world deployment. Likewise, gravitational settling of larger particles (>6 μm) or vibrational noise in unstable environments may introduce drift or bias in measured motion, reducing signal clarity. Conversely, smaller particles (<1 μm), while less prone to sedimentation, are more difficult to visualize using accessible optics. Compared to enzymatically powered Rolosense, FF-Rolosense eliminates the need for RNA substrates, cold storage, or RNase enzymes, greatly improving assay stability and ease of use.

In summary, FF-Rolosense introduces a simple mechanically driven detection paradigm that shifts away from traditional biochemical assays. By coupling passive diffusion with virus-triggered stalling, it enables rapid, sensitive, and portable diagnostics—laying the foundation for broader applications in motion-based biosensing.

## Materials and Methods

### Materials

All oligonucleotides were purchased from Integrated DNA Technologies (IDT), stored at 4 °C (−20 °C for long-term storage), and used without purification. Their sequences, including functional group modifications, are shown in Table S1. Stock solutions were made using Nanopure water (Barnstead Nanopure system, resistivity = 18.2 MΩ), herein referred to as DI water. Aminated silica beads (5 μm) were purchased from Bangs Laboratory (#SA06N). Aminated polystyrene beads (6 μm) were purchased from Spherotech (#AP-60-10). Influenza A/PR/8/34 was purchased from Charles River Laboratories (#10100374). RTube breath condensate collection device was purchased from Respiratory Research (#1025, #3002, and #3001). Thin Au films were generated by using a home-built thermal evaporator system. All motor translocation measurements were performed in Ibidi sticky-slide VI0.4 (Ibidi, #80608) 17 × 3.8 × 0.4 mm channels.

### Microscopy

BF images were acquired on a fully automated Nikon Inverted Research Microscope Eclipse Ti2-E with the Elements software package (Nikon), an automated scanning stage, a 0.50 NA CFI60 Plan Fluor 20× objective, a Prime 95B 25 mm sCMOS (scientific complementary metal-oxide semiconductor) camera for image capture at 16-bit depth, a SOLA SE II 365 Light Engine for solid state white light excitation source, and a perfect focus system used to minimize drift during timelapse. Brightfield timelapse imaging was done using 20× 0.50 NA objective with 5 s per frame rate and an exposure time of 100 ms. All imaging was conducted at room temperature.

### Viruses

UV-inactivated SARS-CoV-2 and human corona (229E, OC43) virus samples at known concentrations were provided by the NIH RADx-Radical Data Coordination Center (DCC) at the University of California, San Diego, and BEI Resources. UV-inactivated SARS-CoV-2 Isolate hCoV-19/USA/PHC658/2021 (Lineage B.1.617.2; Delta Variant), NR-55611, was contributed by Dr. Richard Webby and Dr. Anami Patel. UV-inactivated SARS-CoV-2 Isolate hCoV-19/USA/CA-SEARCH-59467/2021 (Lineage BA.1; Omicron Variant) was contributed by Dr. Aaron Carlin and the UCSD CALM and EXCITE laboratories. Virus samples used in this study have undergone at least one freeze–thaw cycle.

### Thermal Evaporation of Gold Films

A No. 1.5H ibidi glass coverslip (25 × 75 mm) (ibidi #10812) was cleaned by sonication in DI water for 5 min. The sample was then subjected to a second sonication in fresh DI water for 5 min. Finally, the slide was sonicated in 200 proof ethanol (Fischer Scientific #04–355–223) for 5 min and was subsequently dried under a stream of N_2_. The cleaned glass coverslip was then mounted into a home-built thermal evaporator chamber in which the pressure was reduced to 50 × 10^–3^ Torr. The chamber was purged with N_2_ three times, and the pressure was reduced to 1–2 × 10^–7^ Torr by using a turbo pump with a liquid N_2_ trap. Once the desired pressure was achieved, a 3 nm film of Cr was deposited onto the slide at a rate of 0.2 Å s^–1^, which was determined by a quartz-crystal microbalance. After the Cr adhesive layer had been deposited, 6 nm of Au was deposited at a rate of 0.4 Å s^–1^. The Au-coated samples were used within 1 week of deposition.

### Fabrication of DNA Aptamer Monolayers

An Ibidi sticky-Slide VI^0.4^ flow chamber was adhered to the Au-coated slide to produce six channels (17 × 3.8 × 0.4 mm dimensions). Prior to surface functionalization, each channel was rinsed with ∼5 mL of DI water. Next, thiol modified DNA anchor strands were added to each of the channels with 50 μL solution of 1 μM DNA anchor in a 1 M potassium phosphate monobasic (KHPO_4_) buffer. The gold film was sealed by Parafilm to prevent evaporation and the reaction took place overnight at room temperature. After incubation, excess DNA was removed from the channel using a ∼5 mL DI water rinse. To block any bare gold sites and to maximize the hybridization of the DNA aptamer to the DNA anchoring strand, the surface was backfilled with 100 μL of a 100 μM solution of 11-Mercaptoundecyl)­hexa­(ethylene glycol (referred to as SH-PEG) (Sigma-Aldrich #675105) solution in ethanol for 6 h. Excess SH-PEG was removed by a ∼5 mL rinse with ethanol and another ∼5 mL rinse with water. Next, 100 nM of DNA aptamer was added to the surface through hybridization to the DNA anchor in 1× PBS for 12 h. The wells were again sealed with Parafilm to prevent evaporation and the resulting DNA monolayer remained stable for days.

### Synthesis of Azide-Functionalized Particles

Before functionalization with azide, the silica and polystyrene beads were washed to remove any impurities. For the wash, 1 mg of aminated silica beads were centrifuged down for 5 min at 15,000 r.p.m. in 1 mL DI water. Similarly, 1 mg of aminated polystyrene beads were centrifuged down for 10 min at 15,000 r.p.m. in 1 mL DI water with 0.005% of surfactant (Triton-X). The supernatant was discarded, and the resulting particles were resuspended in 1 mL of DI water (silica beads) and 1 mL of DI water with 0.005% Triton-X (polystyrene beads). This was repeated three times and the supernatant was discarded after the final wash. Azide-functionalized particles were then synthesized by mixing 1 mg of aminated silica and polystyrene beads with 1 mg of azido acetic NHS ester (BroadPharm #BP-22467). This mixture was subsequently diluted in 100 μL of dimethyl sulfoxide (DMSO) and 1 μL of a 10× diluted triethylamine stock solution in DMSO. The reaction proceeded overnight for 24 h at room temperature and the azide-modified silica particles were purified by adding 1 μL of DI water and centrifuging down the particles at 15,000 r.p.m. for 5 min. The azide modified polystyrene particles were purified in a similar manner except they were centrifuged for 10 min in 0.005% of Triton-X. The supernatant was discarded, and the resulting particles were resuspended in 1 mL of DI water. This process was repeated seven times, and during the final centrifugation step the particles were resuspended in 100 μL of DI water to yield an azide-modified particle stock. The azide-modified particles were stored at 4 °C in the dark and were used within one month of preparation.

### Synthesis of High-Density DNA Silica and Polystyrene Particles

High-density DNA-functionalized particles were synthesized by adding a total of 5 nanomoles (in 5 μL) of alkyne-modified DNA aptamer stock solution to 5 μL of azide-functionalized particles. The particles and DNA were diluted with 25 μL of DMSO and 5 μL of 2 M triethylammonium acetate buffer (TEAA). Next, 4 μL from a super saturated stock solution of ascorbic acid was added to the reaction as a reducing agent. Cycloaddition between the alkyne-modified DNA and azide-functionalized particles was initiated by adding 2 μL from a 10 mM Cu-TBTA (tris­((1-benzyl-1*H*-1,2,3-triazol-4-yl)­methyl)­amine) stock solution in 55 vol % DMSO (Lumiprobe #21050). The reaction was incubated for 24 h at room temperature on a shaker and the resulting DNA-functionalized particles were purified by centrifugation. Silica particles were centrifuged at 15,000 r.p.m. for 5 min, after which the supernatant was discarded, and the particles were resuspended in 1 mL of a 1× PBS and 10% Triton-X (w/v) solution. This process was repeated seven times, with the particles resuspended in 1 mL 1X PBS only for the fourth to sixth centrifugations. During the final centrifugation, the particles were resuspended in 50 μL of 1X PBS. Polystyrene particles received the same treatment with the exception of being centrifuged at 15,000 r.p.m. for 10 min when purified by centrifugation and being resuspended in 1 mL 1X PBS with 0.005% Triton-X for the fourth to sixth centrifugations. The high-density DNA-functionalized particles were stored at 4 °C and protected from light.

### Breath Condensate Collection

Breath condensate was collected using the R tube breath condensate collection device from Respiratory Research (#1025, #3002, and #3001). The R tube breath condensate collection device consists of three parts: the disposable R tube collector, the cooling sleeve, and the plunger. First, the cooling sleeve was placed in a −20 °C freezer for 15 min. After 15 min, the cooling sleeve was placed on top of the disposable R tube collector and exhaled breath condensate was collected by breathing into the mouthpiece of the R tube collector for 2–5 min. The vapor emerging from the breath was condensed onto the sides of the R tube collector. Following 2–5 min of breathing into the R tube collector, the mouthpiece was removed from the bottom of the R tube collector and the tube was placed on top of the plunger and pushed through it. The exhaled breath condensate was collected into a pool of liquid at the top. The condensed breath was then transferred into an Eppendorf tube and used in creating serial dilutions of the virus samples.

### DNA Particle Incubation with Virus Samples

Before beginning the experiments, known concentration of UV-inactivated virus samples were serially diluted in collected breath condensate to create samples of different virus concentrations. The DNA particles were then incubated with different concentrations of virus samples for 30 min at room temperature. This was done by adding 1 μL of DNA particles (∼800 particles/μL) in 49 μL of breath condensate matrix (±; virus particles). After 30 min of incubation, the DNA particles were added to the aptamer-coated chip which was prewashed with 5 mL of 1X PBS to remove excess unbound DNA aptamer. DNA particles with virus were allowed 1–2 min to settle on the DNA aptamer surface. Particle tracking was achieved through BF imaging by recording a timelapse at five second intervals for 30 min via the Nikon Elements software. The resulting timelapse files were then saved for further analysis.

### Flow Cytometry

For each DNA particle functionalized with a different aptamer sequence, one 50 μL aliquot representing the experimental condition and one 50 μL aliquot representing the negative control was generated. For the experimental condition, the high-density DNA-functionalized particles were incubated with a solution containing a FAM-tagged DNA complement in 1X PBS. Specifically, 5 μL of DNA particles and 5 μL of 1 μM FAM-tagged DNA complement solution were added to 40 μL of 1X PBS. For the negative control, the high-density DNA-functionalized particles were incubated in only 1X PBS. Specifically, 5 μL of DNA particles was added to 45 μL of 1X PBS overnight. All aliquots were wrapped in aluminum foil and left on the VWR Incubating Mini Shaker at a speed of 340 r.p.m for the DNA particles to incubate in solution overnight (12 h). After the incubation period, the DNA particles were washed twice with 1 mL of 1X PBS and then a final time with 100 μL 1X PBS. All washes were performed in the centrifuge at 15,000 r.p.m for 5 min. In the final step, the DNA particles were resuspended in 50 μL 1X PBS. A triplicate set of 50 μL aliquots were generated for both the negative control and experimental aliquot of each DNA particle type. For each aliquot in the triplicate set, 12 μL of the washed DNA particles was added to 38 μL 1X PBS. Each aliquot was then run through the Cytoflex in the CD69 configuration with the following gain settings: FSC = 22, SSC = 43, and GFP = 164. For the binding affinity studies with the VLPs, three 50 μL aliquots representing each concentration in the experimental condition, and three 50 μL aliquots of the buffer representing the negative control were generated. For both the experimental condition and the negative control, 5 μL of DNA particles was added to each 50 μL aliquot. All aliquots were wrapped in aluminum foil and left on the VWR Incubating Mini Shaker at a speed of 480 r.p.m for the DNA particles to incubate in solution for 30 min. After the incubation period, each aliquot was then run through the Cytoflex in the CD69 configuration with the following gain settings: FSC = 22, SSC = 43, and GFP = 2000. For all flow cytometry studies, the flow rate was set to 20 μL/min and each run was set to a stopping rule of 30 μL or 5000 events. The data was then analyzed with FlowJo_v10.10.0, where a polygon gate was applied to the FSC-A vs SSC-A scatterplots that represented events detected in the negative control aliquot for each DNA particle type. The gates were applied to isolate only populations representing 5 μm silica particles for future analysis. The same gates applied to the negative control scatterplots of each DNA particle type were also applied to the experimental condition scatterplots of each DNA particle type. Using these gates, histograms representing the FAM signal from the FAM-tagged DNA or the GFP signal from the VLPs (indicated as GFP-A in FlowJo) exhibited by the events within the isolated populations and the median FAM or GFP signal exhibited by each population were obtained. Statistical analyses were performed on the median values of FAM or GFP signal from the negative control and experimental condition of each DNA particle type in GraphPad v. 9.1.0.

### Image Processing and Particle Tracking

Image processing and particle tracking was performed in Fiji (ImageJ) as well as Python. The Bioformats toolbox in Fiji (ImageJ) enabled direct transfer of Nikon Elements image files (*.nd2) into the Fiji (ImageJ) environment where all image/video processing was performed. Particle tracking was performed using the 2D/3D particle tracker from the Mosaic plugin in Fiji (ImageJ)^16^ in which we generated .csv files with particle trajectories that were used for further analysis. The algorithms for processing the data for particle trajectories and net displacements were performed on Python v. 3.7.4. Calculation of drift correction was adapted from trackpy (github.com/softmatter/trackpy). Full Python script from brightfield acquisition data can be found at https://github.com/spiranej/particle_tracking_. Statistical analyses were performed in GraphPad v. 9.1.0. One-way ANOVA tests were used to analyze the net displacements of the particle trajectories in each experimental condition and the median fluorescence signals exhibited in the flow cytometry assays.

### DeepSPT Analysis of DNA Particle Diffusion

To analyze the diffusional behavior of DNA particles, we used DeepSPT, a deep learning-based single-particle tracking framework developed by Kaestel-Hansen et al.^33,38^ The adapted code used in this work is available at github.com/JKaestelHansen/DNAmotor_analysis.

#### Temporal Segmentation

The first DeepSPT module performs frame-by-frame segmentation of particle motion. While the original model classifies trajectories into four diffusion states, we collapsed this into a binary classification: “Free” (directed and Brownian motion) and “Restricted” (confined and subdiffusive motion), consistent with the expected bimodal behavior of our system. This segmentation allows us to determine whether a particle was persistently free, persistently restricted, or transitioned between states. We also annotate the duration of restricted motion per particle trajectory.

#### Feature Extraction

The second DeepSPT module computes 30 diffusional and temporal features per trajectory, including net displacement, diffusion coefficient, directionality, time spent restricted, and number of changepoints. These features serve as both a descriptive metric set and a fixed-length input for machine learning classification tasks.

#### Classification of Viral Exposure

The third module classifies trajectories based on viral exposure using a task-specific approach. For binary classification (virus vs no virus), we used logistic regression trained on tracks from 10^6^ copies/mL SARS-CoV-2 BA.1 (virus class) and no-virus samples. Two additional criteria were applied as fail-safes: (1) tracks with net displacement <15 μm were labeled “virus” and (2) tracks restricted for ≥50 frames were designated “persistently restricted.”

For multiclass classification by viral concentration, we used an ensemble of three random forest classifiers (100 trees each). Maximum tree depths were adjusted based on the classification task:

- All concentrations: max depths = (6, 7, 8)
- 10^4^ copies/mL vs no virus: (3, 4, 5)
- 10^3^ copies/mL vs no virus: (9, 10, 11)
- All other cases: (7, 8, 9)

Final predictions were based on majority voting. Classifier performance was evaluated using 10-fold cross-validation with shuffling. To address class imbalance, random oversampling of the minority class was used. All models were implemented using **scikit-learn** version 1.2.0.^39^

## Supplementary Material



## Data Availability

Source statistical data are provided with this paper. Additional data sets generated are available from the corresponding author on reasonable request. Python scripts from brightfield acquisition data regarding net displacements and particle ensemble trajectories can be found at https://github.com/spiranej/particle_tracking_.

## Notes

Detailed protocols for particle and chip functionalization, buffers, and exhaled breath condensate collection; controls demonstrating the physical basis of the assay—ionic-strength dependence of Brownian motion, immobilization without particle-bound aptamers, and long-term particle mobility without virus; stage-tilt (gravity) effects and acquisition timeline; automated particle-tracking workflow; influenza A detection using 6 μm polystyrene particles; NUPACK secondary-structure predictions for aptamers with varying spacers; quantification of particle DNA loading by saturation hybridization/flow cytometry and comparison of surface functionalization; validation of PEG36-aptamer affinity to Omicron pseudovirus; FF-Rolosense detection with PEGylated aptamers (BA.1) and variant testing in EBC (BA.5.1, XBB.1.5); matrix dependence of binding across PBS dilutions vs EBC; Roloscope specifications and build details (PDF)
