# Supplementary material for: Fuel-Free Rolosense: Viral Sensing Using Diffusional Particle Tracking
Source: ACS Sens. 2025 Nov 5;10(11):8542–53. doi: 10.1021/acssensors.5c02311 (PMC12670994; doi:10.1021/acssensors.5c02311)
Supplement: Supplementary file 1 [file se5c02311_si_001.pdf]

Supplementary Information for
Fuel-free Rolosense: viral sensing using diffusional particle tracking

Selma Piranej[1], Krista Jackson[1,2], Luona Zhang[1], Jacob Kaestel Hansen[3,4,5], Frank Sommerhage[6], David DeRoo[6], Nikos S. Hatzakis[3,4,5], Khalid Salaita[1,2] *

---

[1]Department of Chemistry, Emory University, Atlanta, GA 30322 (USA). [2]Wallace H. Coulter Department of Biomedical Engineering, Georgia Institute of Technology and Emory University, Atlanta, GA 30322 (USA). [3]Department of Chemistry, University of Copenhagen, Copenhagen 2100 (Denmark). [4]Center for 4D cellular dynamics, University of Copenhagen, Copenhagen 2100 (Denmark). [5]Novo Nordisk Center for Optimised Oligo Escape and Control of Disease, University of Copenhagen, Copenhagen 2100 (Denmark). [6]Primordia Biosystems, Costa Mesa, CA 92626 (USA).

*Correspondence should be addressed to: k.salaita@emory.edu

Table of Contents

Supplementary Table 1 3

Supplementary Table 1 4

Supplementary Figure 1. Ionic strength modulates Brownian motion of DNA particles via electrostatic repulsion. 5

Supplementary Figure 2. Immobilization of particles in the absence of particle-bound aptamers. 6

Supplementary Figure 3. DNA particle diffusivity remains stable over time in the absence of virus 7

Supplementary Figure 4. Effect of gravity on DNA aptamer coated particles through tilt. 8

Supplementary Figure 5. Timeline for FF-Rolosense acquisition. 9

Supplementary Figure 6. Particle tracking workflow. 10

Supplementary Figure 7. Detection of influenza A viruses using 6 μm polystyrene particles. 11

Supplementary Figure 8. Secondary structure predictions of Delta-variant aptamers with varying spacer lengths 12

Supplementary Figure 9. Quantifying DNA loading on particles by saturation hybridization and flow cytometry. 13

Supplementary Figure 10. The effect of aptamer length on surface functionalization. .. 14

Supplementary Figure 11. Validation of PEG-aptamer binding affinity to SARS-CoV-2 Omicron pseudovirus 15

Supplementary Figure 12. Detection of SARS-CoV-2 BA.1 using PEGylated aptamer. 17

Supplementary Figure 13. Detection of SARS-CoV-2 BA.5.1 and XBB.1.5 in exhaled breath condensate using FF-Rolosense. 18

Supplementary Figure 14. Binding of PEG36–aptamer particles across different matrices. 19

Supplementary Figure 15. Roloscope specifications. 20

Supplementary Table 1: Oligonucleotide sequence design

ID	Sequences (5'-3')
DNA Anchor	/5AmMC6/GAGAGAGATGGGTGCTTTTTTTTTTTTTTT/3ThiolMC3-D/
Omicron Particle Aptamer[1]	5Hexynyl/TTTTTATCCAGAGTGACGCAGCACCGACCTTGTGCTTTGGGAGTGC TGGTCCAAGGGCGTTAATGGACACGGTGGCTTAGT
Omicron Surface Aptamer	GCACCCATCTCTCTCATCCAGAGTGACGCAGCACCGACCTTGTGCTTTGGG AGTGCTGGTCCAAGGGCGTTA ATGGACACGGTGGCTTAGT
Influenza A Particle Aptamer[2]	/5Hexynyl/TTTTTGGCAGGAAGACAAACAGCCAGCGTGACAGCGACGCG TAGGGACCGGCATCCGCGGGTGGTCTGTGGTGCTGT
Influenza A Surface Aptamer	GCACCCATCTCTCTCGGCAGGAAGACAAACAGCCAGCGTGACAGCGAC GCGTAGGGACCGGCATCCGCGGGTGGTCTGTGGTGCTGT
T20 Particle Aptamer (Delta)[3]	/5Hexynyl/TTTTTTTTTTTTTTTTTTTTCCCATGGTAGGTATTGCTTGGTAGGGA TAGTGGG
T20 Surface Aptamer (Delta)	GCACCCATCTCTCTCTTTTTTTTTTTTTTTTTTTTCCCATGGTAGGTATTGCTTGG TAGGGATAGTGGG
T40 Particle Aptamer (Delta)	/5Hexynyl/TTTTTTTTTTTTTTTTTTTTTTTTTTTTTTTTTTTTTTTTCCCATGGTAGGT ATTGCTTGGTAGGGATAGTGGG
T40 Surface Aptamer (Delta)	GCACCCATCTCTCTCTTTTTTTTTTTTTTTTTTTTTTTTTTTTTTTTTTTTTTTTTCCC ATGGTAGGTATTGCTTGGTAGGGATAGTGGG
PEG36 Particle Aptamer (Delta)	/5Hexynyl//iSp18//iSp18/ /iSp18//iSp18//iSp18/ /iSp18/CCCATGGTAGGTATTGC TTGGTAGGGATAGTGGG
PEG36 Surface Aptamer (Delta)	GCA CCC ATC TCT CTC /iSp18//iSp18//iSp18/ /iSp18//iSp18//iSp18/ CCCATGGTA GGTATTGCTTGGTAGGGATAGTGG G
Omicron PEG36 Particle Aptamer	/5Hexynyl//iSp18//iSp18/ /iSp18/ATCCAGAGTGACGCAGCACCGACCTTGTGCTT TGGGAGTGCTGGTCCAAGGGCGTTAATGGACACGGTGGCTTAGT
Omicron PEG36 Surface Aptamer	GCA CCC ATC TCT CTC /iSp18//iSp18/A TCCAGAGTGACGCAGCACCGACCT TGTGCTTTGGGAGTGCTGGTCCAAGGGCGTTAATGGACACGGTGGCTTAGT
RSV A Particle Aptamer[4]	/5Hexynyl/TTTTTAGTGCGGTGAGCCGTCGGACATACAAATAC
RSV A Surface Aptamer	GCACCCATCTCTCTCAGTGCGGTGAGCCGTCGGACATACAAATAC
Omicron Particle Aptamer FAM complement	/56-FAM/ACTAAGCCACCGTGTCCATTAACGCCCTTGGACCAG
Particle Aptamer (Delta) FAM complement	/56-FAM/CCCACTATCCCTACCAAGCAATACCTACCATGGG
Scrambled Particle DNA	/5Hexynyl/TTTTTGGAGCGTTCAGCTTGTTTACTGACCCAAGGTTCGTTGACCGA CTGAGACTGTGT AGTATCGGTCTGTGGAAGGGAA
PEG36 Scrambled Particle DNA	/5Hexynyl//iSp18//iSp18//iSp18/GGAGCGTTCAGCTTGTTTACTGACCCAAGGTTC GTTGACCG ACTGAGACTGTGTAGTATCGGTCTGTGGAAGGGAA

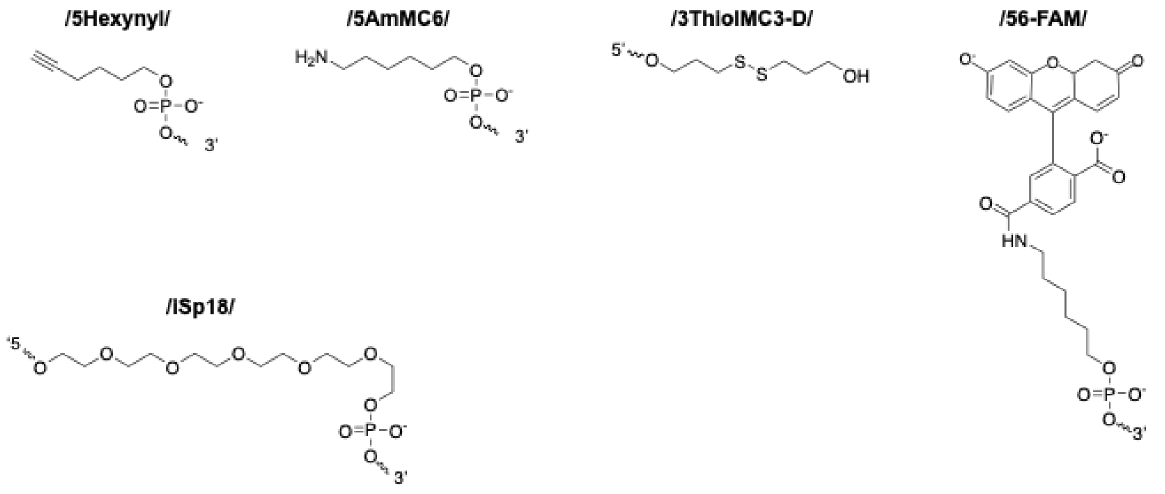

Supplementary Table 1. Table summarizing the sequences of oligonucleotides used in the design of the FF-Rolosense assay displayed in a 5’ to 3’ orientation. The 5’, 3’, and internal DNA modifications are indicated in the table and illustrated below it.

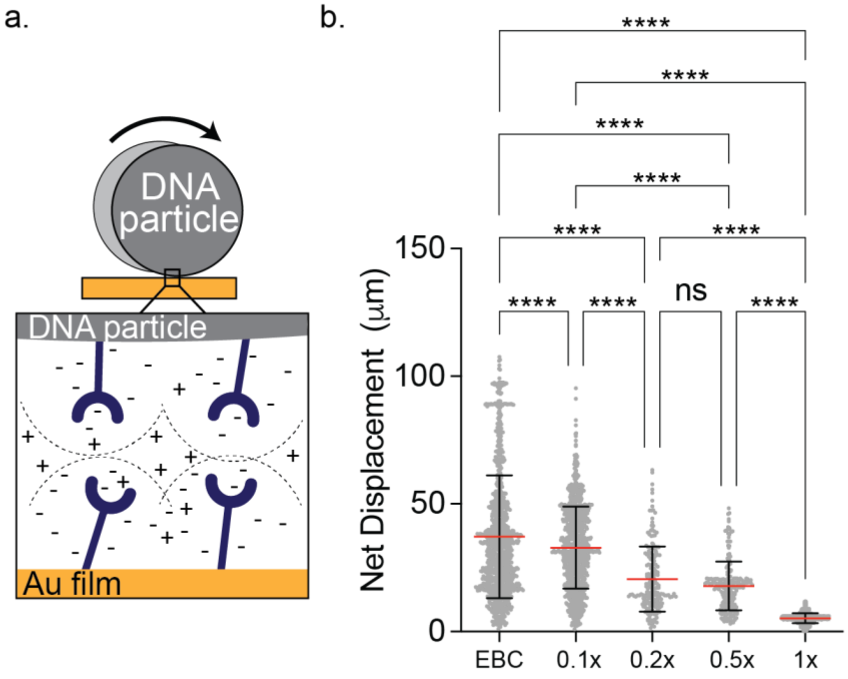

Supplementary Figure 1. Ionic strength modulates Brownian motion of DNA particles via electrostatic repulsion. a, Schematic of FF-Rolosense illustrating electrostatic repulsion between DNA-functionalized particles and surface in low ionic strength media. The negatively charged DNA coatings on both the particle and the gold substrate create repulsive forces that enable lateral diffusion without nonspecific adhesion. **b,** Net displacement of DNA particles in virus-free samples prepared in either exhaled breath condensate (EBC) or phosphate-buffered saline (PBS) at varying ionic strengths (0.1x, 0.2x, 0.5x, 1x). EBC and highly diluted PBS support high particle mobility, whereas increasing ionic strength leads to lower motility due to reduced electrostatic screening. Each condition represents pooled data from >200 tracked particles. Red lines denote mean values; error bars represent standard deviation. ns is not significant; **** indicates $p < 0.0001$.

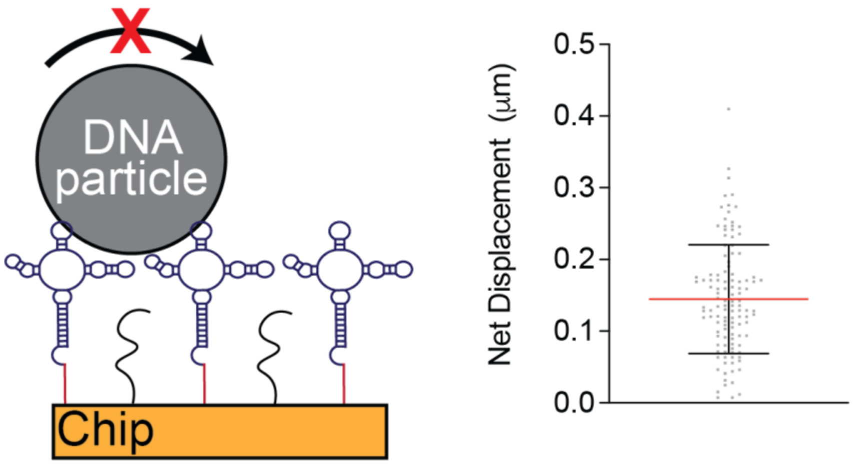

Supplementary Figure 2. Immobilization of particles in the absence of particle-bound aptamers. Schematic and corresponding displacement data showing that particles lacking aptamers fail to roll when placed on surfaces functionalized with DNA aptamers. Net displacement of 118 tracked particles remains near zero, confirming that multivalent interactions are required on both particle and surface for motion to occur.

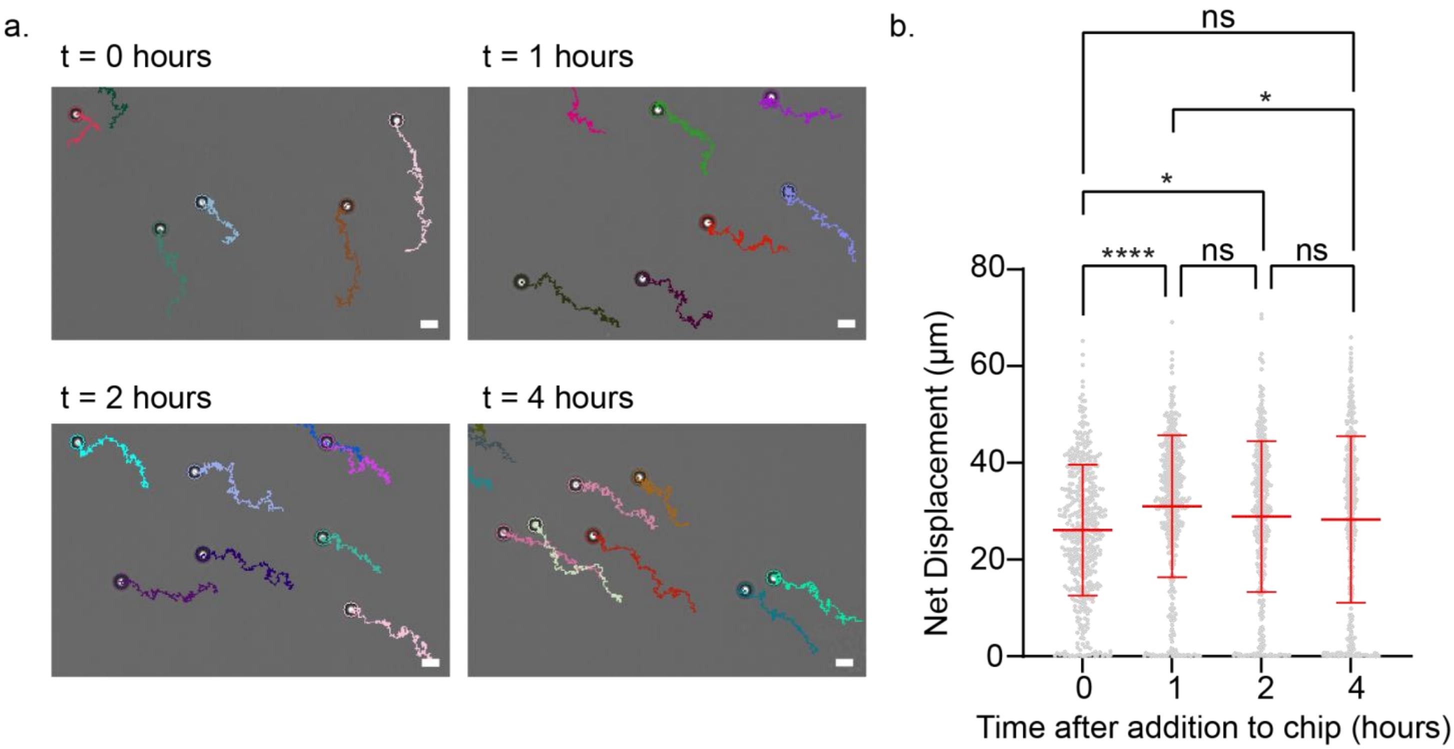

Supplementary Figure 3. DNA particle diffusivity remains stable over time in the absence of virus. a, Representative brightfield images with trajectory overlays showing DNA particle motion at 0, 1, 2, and 4 hours after loading onto the functionalized chip. The trajectories are from 30 min timelapses recorded at each time point. Particles were incubated in virus-free exhaled breath condensate. No significant change in particle mobility was observed over time over 30 min timelapses. Scale bars: 10 μm. **b,** Plot of net displacement for particles tracked at each time point. Each point represents a single particle; red lines and error bars indicate the mean and standard deviation. Results indicate that particle motion remains robust and consistent for up to 4 hours, confirming assay stability in the absence of viral targets. **** $p < 0.0001$, * $p < 0.05$, ns is not significant.

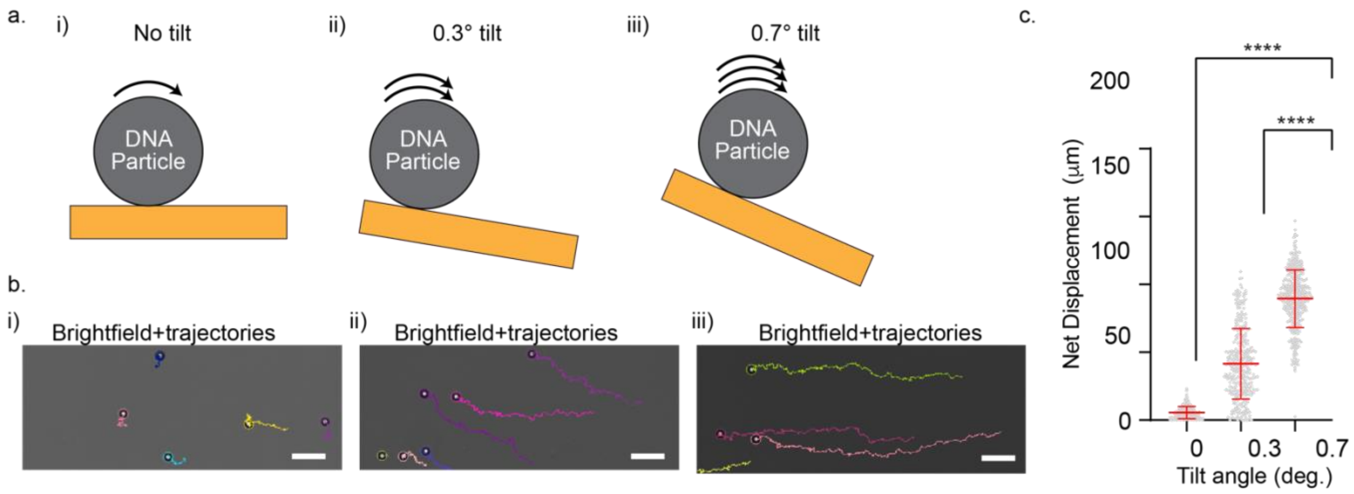

Supplementary Figure 4. Effect of gravity on DNA aptamer coated particles through tilt. a, Schematic depicting particle motion under three tilt conditions: (i) 0°, (ii) 0.3°, and (iii) 0.7°. Increasing tilt introduces a gravitational component that biases particle movement. **b,** Representative brightfield images with trajectory overlays for each tilt condition. At 0°, particles exhibit random Brownian motion; increasing tilt causes directional drift. Scale bars: 10 µm. **c,** Quantification of net displacement for particles under each tilt angle. Red lines and error bars represent the mean and standard deviation, respectively. Over 100 particles were analyzed per condition. **** indicates $p < 0.0001$

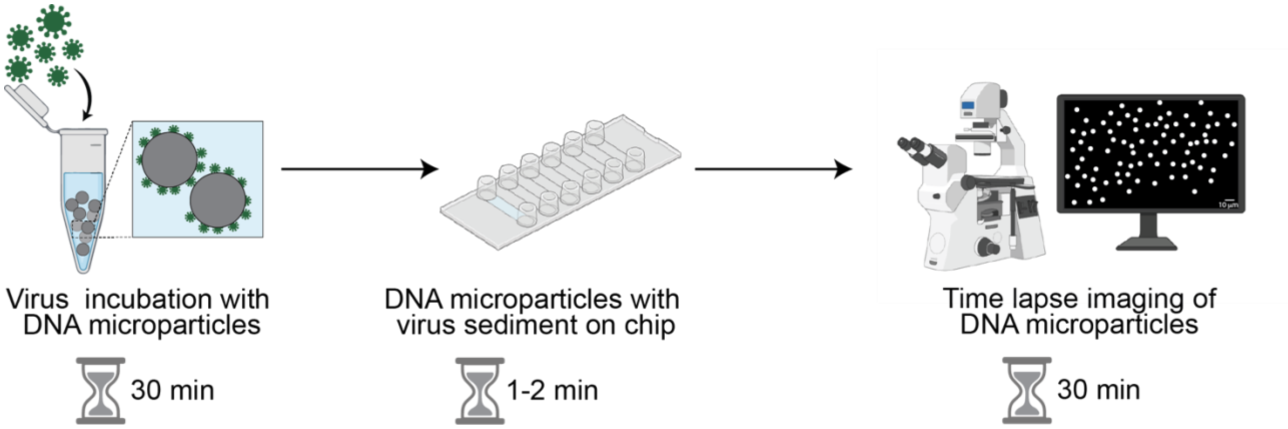

Supplementary Figure 5. Timeline for FF-Rolosense acquisition. Virus (or control) is mixed with 5 µm DNA-aptamer coated silica microparticles and incubated off-chip for 30 min at room temperature. The suspension is then loaded into the functionalized chip; particles settle in ~1–2 min. Brightfield time-lapse imaging is started immediately and acquired for 30 min, during which virus-induced stalling of particle motion is quantified.

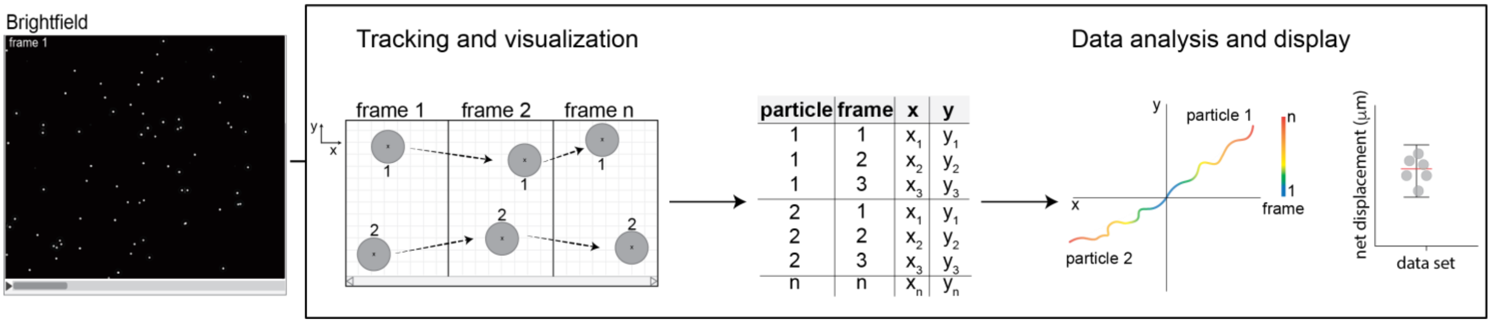

Supplementary Figure 6. Particle tracking workflow. Illustration of the workflow for tracking and analyzing particle motion in brightfield microscopy. The brightfield image (left) shows particles at frame or time point 1. The tracking process identifies and visualizes particle trajectories across multiple frames (middle) and records positional data (x, y) for each particle over time. The positional data is then analyzed to compute and visualize displacement trajectories (right), with color indicating frame number, and statistical representations of net displacements are displayed for the dataset.

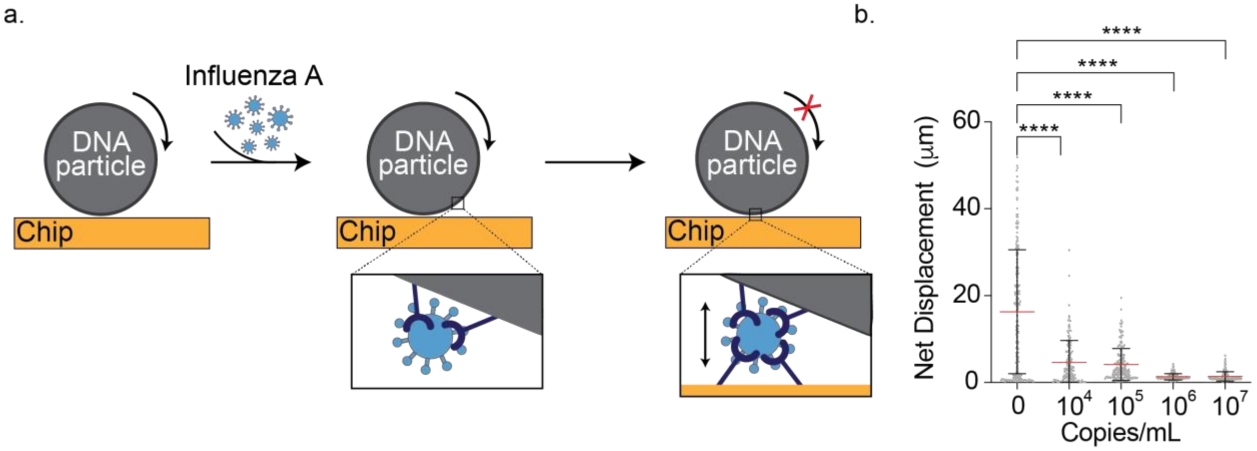

Supplementary Figure 7. Detection of influenza A viruses using 6 μm polystyrene particles. **a,** Schematic of FF-Rolosense detection of Influenza A virus. DNA-functionalized 6 μm polystyrene particles diffuse freely across the aptamer-coated chip in the absence of virus. Upon exposure to Influenza A, multivalent interactions between viral surface proteins and aptamers on both the particle and surface lead to mechanical stalling of the particle. **b,** Net displacement of particles following 30-minute incubation with varying concentrations of UV-inactivated Influenza A virus spiked into exhaled breath condensate. Over 100 particles were analyzed per condition. Results confirm effective motion-based detection of Influenza A using an alternate particle type and size. Red lines and error bars represent the mean and standard deviation. **** indicates $p < 0.0001$.

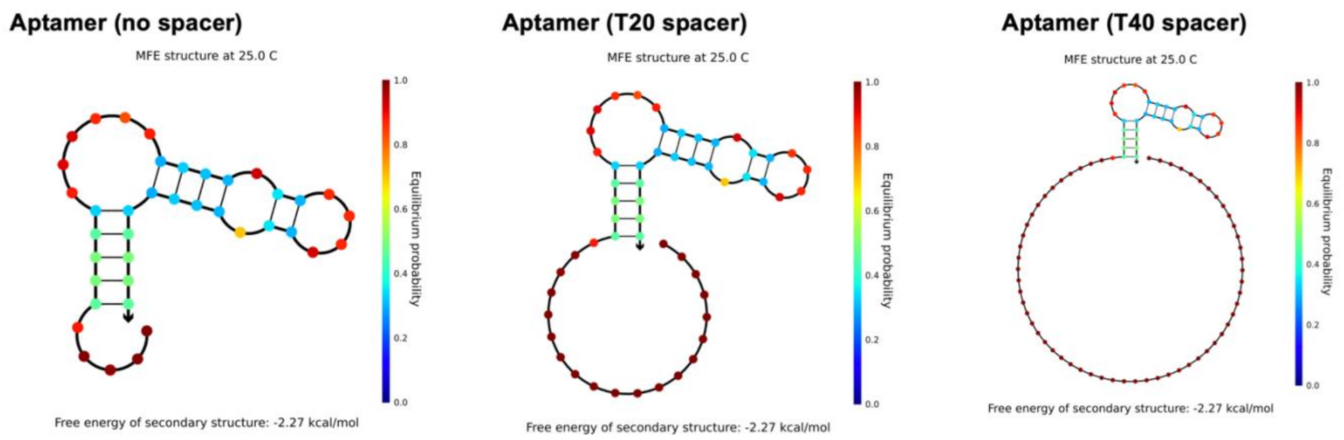

Supplementary Figure 8. Secondary structure predictions of Delta-variant aptamers with varying spacer lengths. Secondary structures of aptamers with high affinity to Delta variant were predicted using NUPACK (http://www.nupack.org/). For aptamers with T20 and T40 spacers using the following conditions: 25˚C, 0.137 M Na^{+}. Arrows represent the 3' end of the aptamer. Nucleotides are color-coded by their equilibrium probability (legend on right).

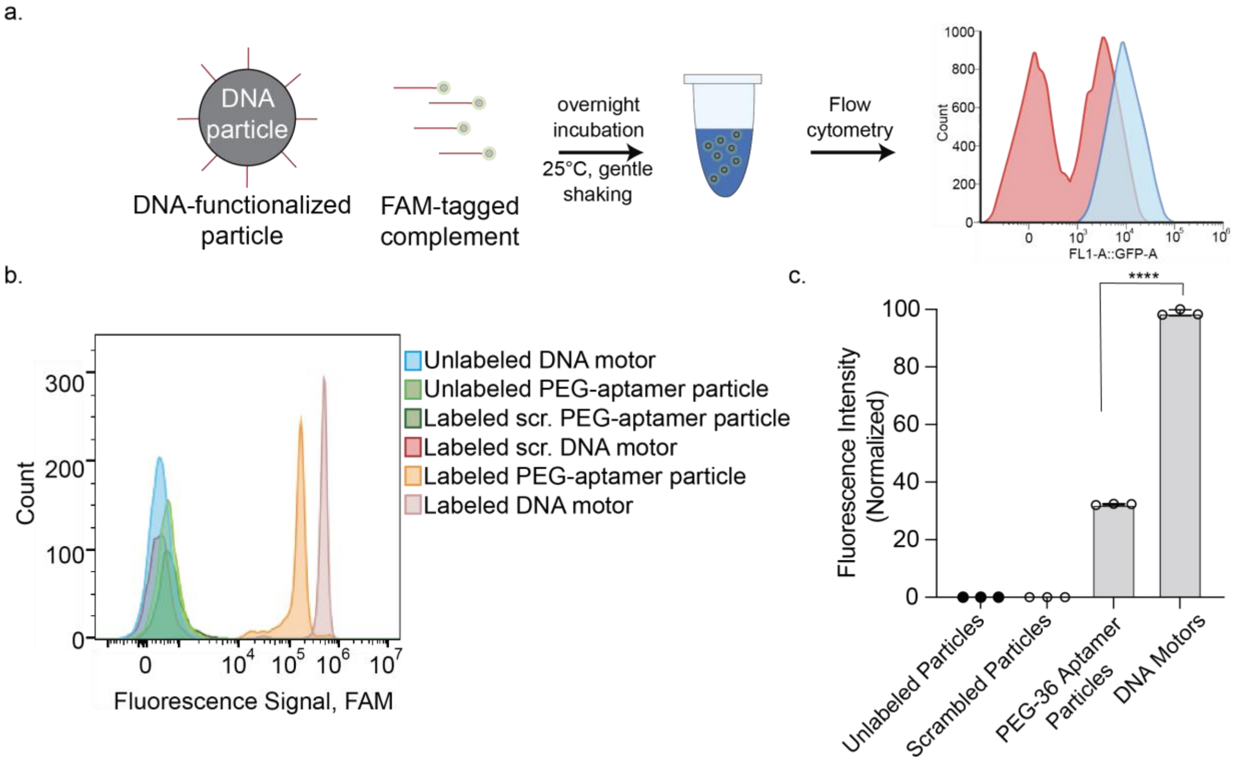

Supplementary Figure 9. Quantifying DNA loading on particles by saturation hybridization and flow cytometry. a, Assay schematic. DNA-functionalized 5-μm silica particles were incubated with excess FAM-tagged complementary oligo (overnight, 25 °C, gentle shaking), washed, and analyzed by flow cytometry. **b,** Representative histograms of FAM signal for unlabeled controls (blue and light green), scrambled-sequence controls (dark green and red), and FAM-hybridized samples (PEG-36 aptamer particles and DNA motors; orange/pink). A rightward shift indicates higher per-particle fluorescence. **c,** Normalized particle fluorescence across conditions: unlabeled and scrambled controls (baseline), PEG-36 aptamer particles and DNA-motor particles. Benchmarking to the independently established density of DNA-motor particles **(**~7 × 10^6 strands per 5-μm bead) indicates that PEG36-aptamer carries ~30–35% of the reference loading**,** corresponding to ~2–2.5 × 10^6 strands per bead. Bars, mean; points, replicates; **** $p < 0.0001$.

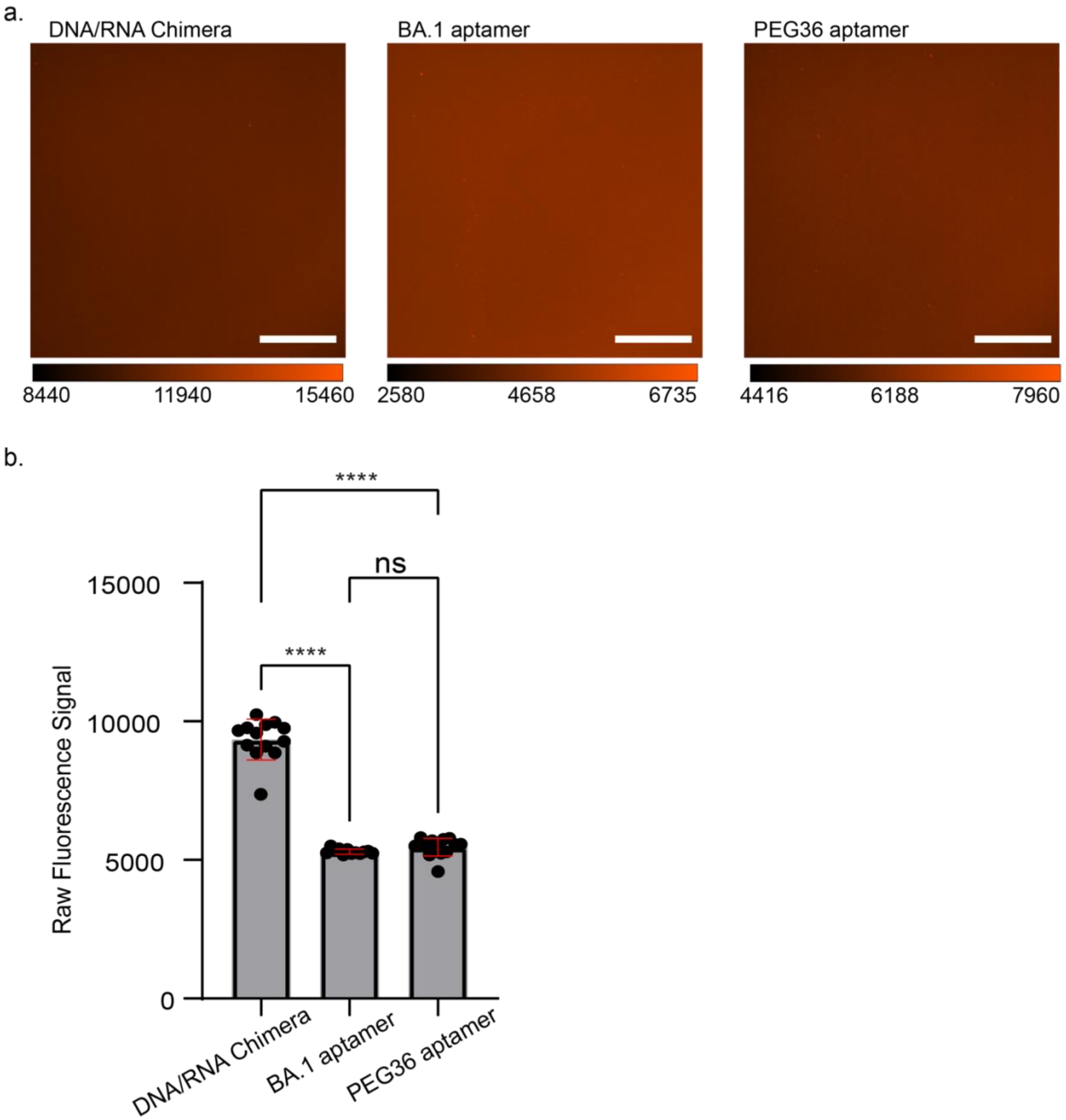

Supplementary Figure 10. The effect of aptamer length on surface functionalization. a, Representative images indicating the relative fluorescence intensities of DNA/RNA chimera, the BA.1 aptamer, and the PEG36 aptamer, all fluorescently tagged with Cy3. The scale bar is 25 μm. **b,** Bar graph of the mean fluorescence intensity obtained from imaging the different surfaces (performed in triplicate) indicate that the 81 bp BA.1 aptamer and the 76 bp PEG36 aptamer exhibit significantly less relative Cy3 signal (**** indicates $p<0.0001$) than the 32 bp DNA/RNA chimera.

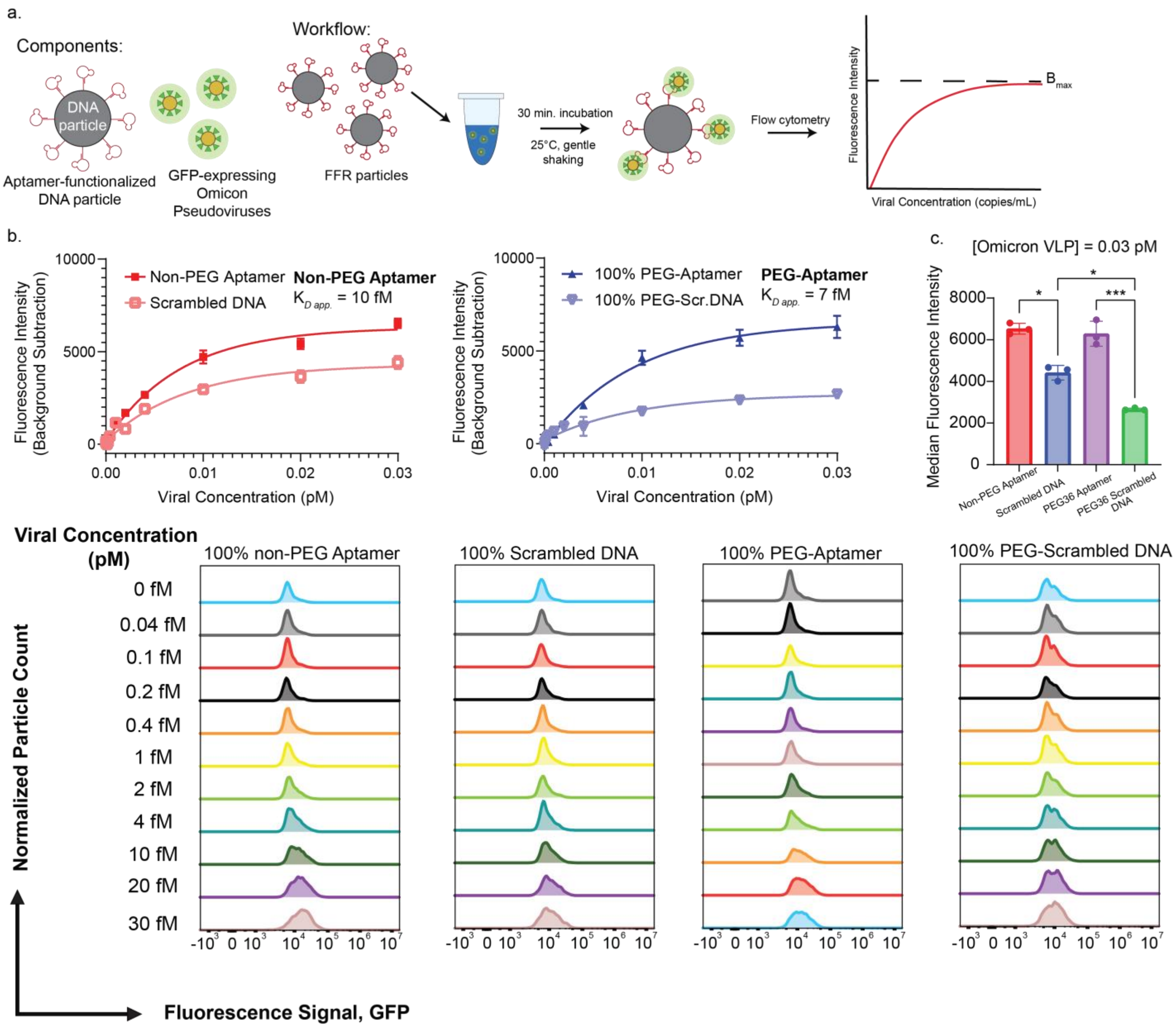

Supplementary Figure 11. Validation of PEG-aptamer binding affinity to SARS-CoV-2 Omicron pseudovirus. a, Schematic of the solution-phase binding assay. DNA particles functionalized with either PEGylated or non-PEGylated aptamers were incubated with GFP-expressing Omicron pseudoviruses for 30 minutes at 25°C, followed by flow cytometry to quantify binding via GFP signal. **b,** Binding curves showing fluorescence intensity as a function of viral concentration for (left) non-PEGylated aptamer vs. scrambled DNA control, and (right) PEG-aptamer vs. PEG-scrambled DNA. Apparent dissociation constants ($K_{d,\ app}$) were 10 fM and 7 fM for the non-PEG and PEG versions, respectively. Apparent K_d values were obtained in 1x PBS (0.55 mM $MgCl_2$) by nonlinear least-squares fitting of the per-series min–max normalized binding data to a Hill model. **c,** Median fluorescence intensity at 0.03 pM virus concentration. PEG-aptamer

shows significantly higher binding than scrambled controls, confirming target specificity. **d,** Flow cytometry histograms of fluorescence signal distributions across increasing viral concentrations (0–30 fM) for each condition. PEGylation of the aptamer increases signal amplitude and improves binding sensitivity when compared with the PEG36-scrambled DNA. Data are representative of three independent replicates.

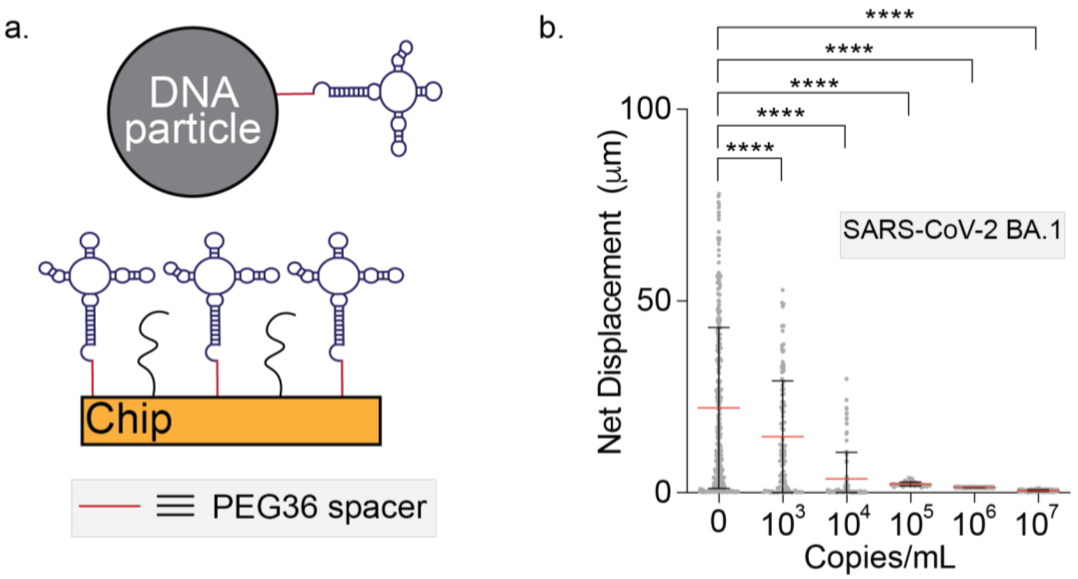

Supplementary Figure 12. Detection of SARS-CoV-2 BA.1 using PEGylated aptamer. a, Schematic of FF-Rolosense in which DNA particles and chip surfaces are functionalized with a PEG_{36}-modified aptamer targeting the receptor-binding domain (RBD) of SARS-CoV-2 BA.1. The flexible PEG spacer (~12 nm) enhances aptamer accessibility and packing density. **b,** Net displacement of DNA particles after 30-minute incubation with varying concentrations of UV-inactivated SARS-CoV-2 BA.1 (10^3–10^7copies/mL) in EBC. Increased viral concentrations correspond to reduced particle mobility. The error bars and the red lines represent the standard deviation and the mean of the distribution, respectively. Experiments were run in triplicate. **** indicates p<0.0001

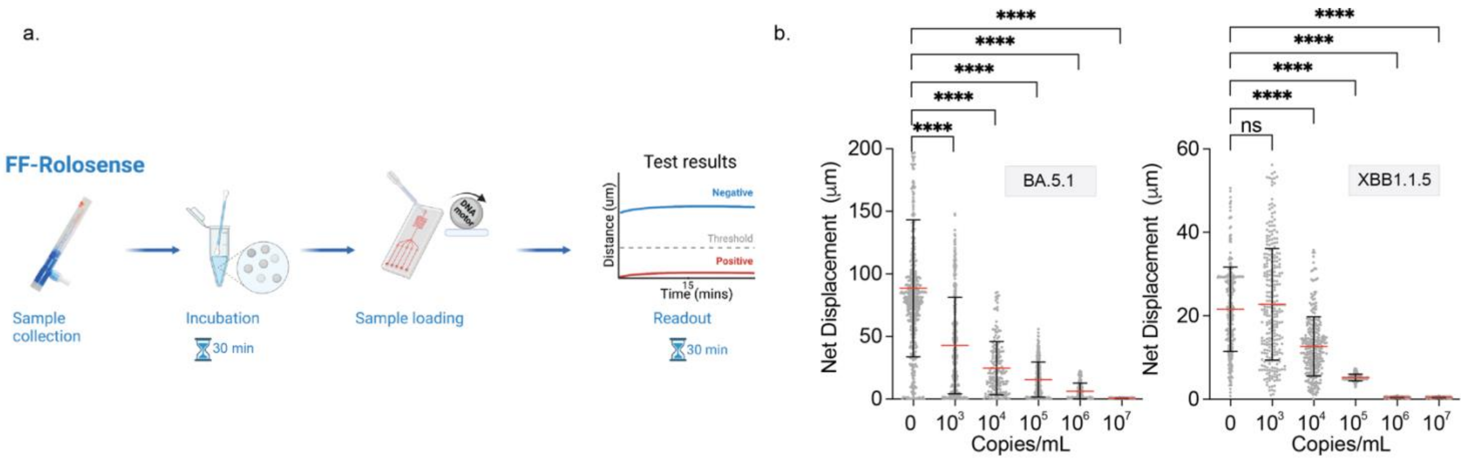

Supplementary Figure 13. Detection of SARS-CoV-2 BA.5.1 and XBB.1.5 in exhaled breath condensate using FF-Rolosense. a, Workflow FF-Rolosense assay including sample collection and incubation with the DNA microparticles for 30 mins at room temp followed by imaging. **b,** Plots of net displacement for over 100 motors incubated with different concentrations of UV-inactivated SARS-CoV-2 BA.5.1 and XBB.5.1 samples spiked in breath condensate. The error bars and the red lines represent the standard deviation and the mean of the distribution, respectively. Experiments were run in triplicate. ns and **** indicate not statistically significant and $p<0.0001$, respectively.

a.

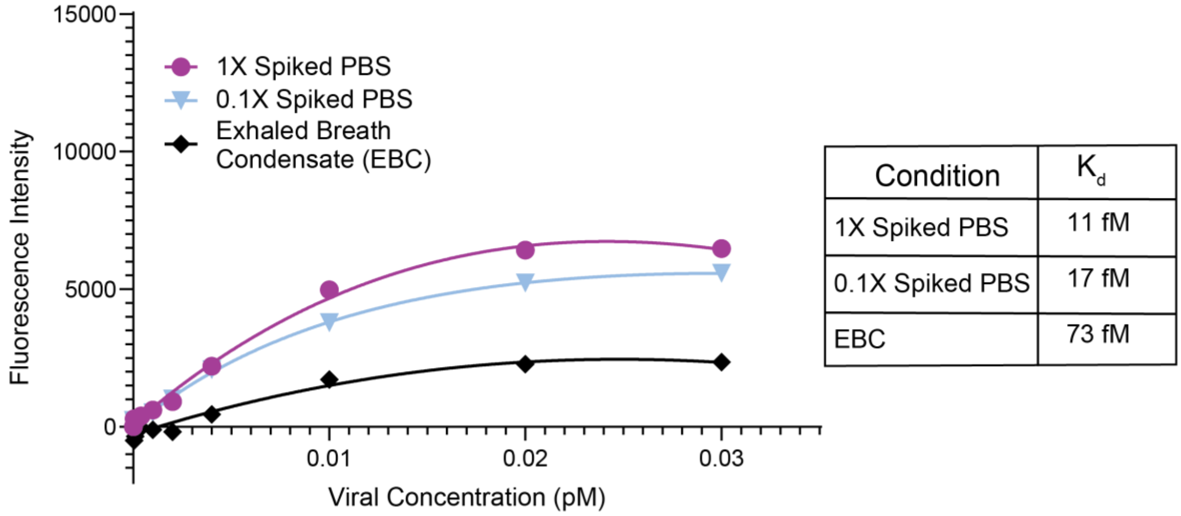

Condition	K_d
1X Spiked PBS	11 fM
0.1X Spiked PBS	17 fM
EBC	73 fM

Supplementary Figure 14. Binding of PEG36–aptamer particles across different matrices. a, Background-subtracted median fluorescence (points) was fit by nonlinear regression to a one-site Hill model with slope fixed to 1 $(Y = B_{\max}X/(K_d + X))$. $B_{\max}$ was first estimated from the 1x PBS series and then held constant when fitting 0.1x PBS and EBC (no per-series normalization). Best fit K_d values are 11 fM (1x PBS), 17 fM (0.1x PBS), and 73 fM (EBC).

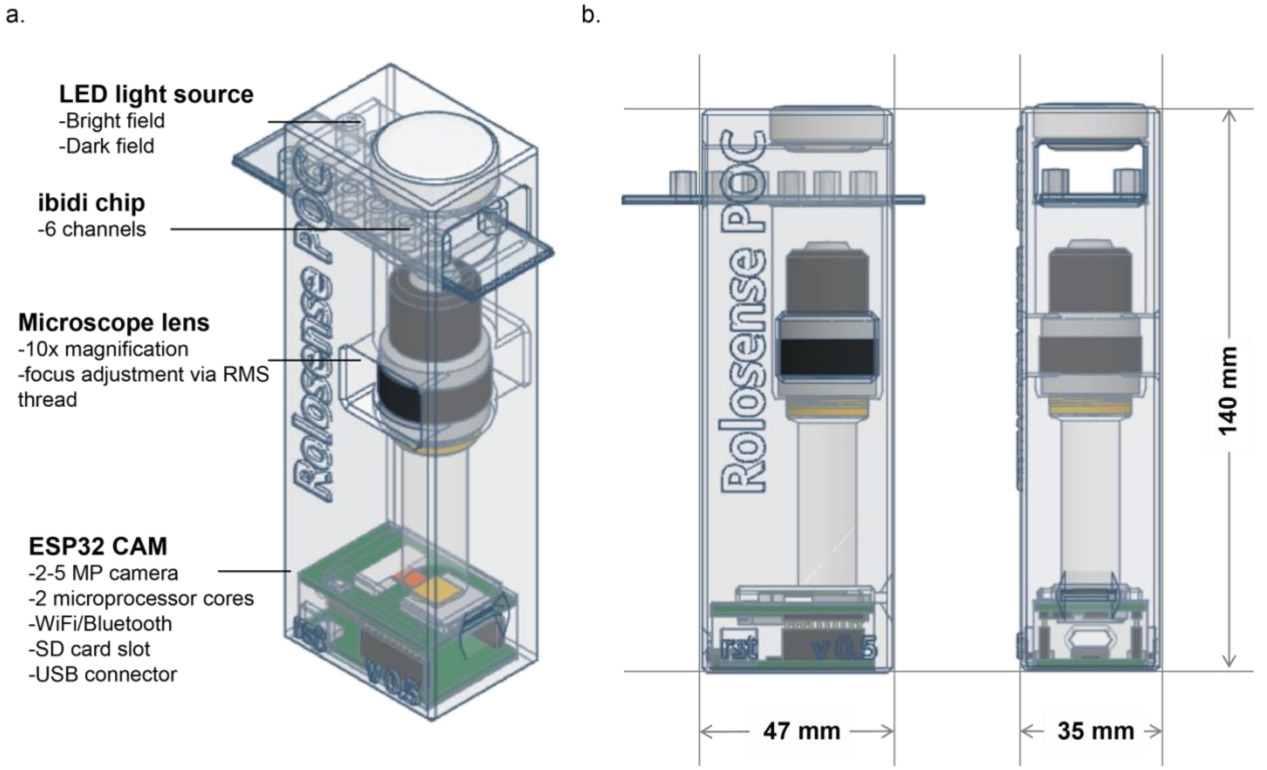

Supplementary Figure 15. Roloscope specifications. a, Integrated components and **b,** measurements of *Roloscope* imager. Roloscope utilizes the ESP32-CAM-MB module which integrates a dual-core processor, providing substantial computational capability with low power consumption. The processor can run up to 240 MHz, enabling automated image capture, processing, and on-chip particle tracking. The integrated OV2640 camera sensor can capture 2-megapixel images and supporting an output image format of JPEG, BMP, and GRAYSCALE. It further features an integrated LED light source and a 10x microscope lens. Roloscope supports Wi-Fi (IEEE 802.11 b/g/n/e/i) enabling wireless communication and data transfer functionalities.

References:

(1) Song, Y.; Song, J.; Wei, X.; Huang, M.; Sun, M.; Zhu, L.; Lin, B.; Shen, H.; Zhu, Z.; Yang, C.; Discovery of Aptamers Targeting the Receptor-Binding Domain of the SARS-CoV-2 Spike Glycoprotein. *Analytical Chemistry* **2020**, 92, 9895–9900. DOI: 10.1021/acs.analchem.0c01394
(2) Lai, H. C.; Wang, C. H.; Liou, T. M.; Lee, G. B. Influenza A virus-specific aptamers screened by using an integrated microfluidic system. *Lab Chip* **2014**, *14* (12), 2002-2013. DOI: 10.1039/c4lc00187g
(3) Schmitz, A.; Weber, A.; Bayin, M.; Breuers, S.; Fieberg, V.; Famulok, M.; Mayer, G. A SARS-CoV-2 Spike Binding DNA Aptamer that Inhibits Pseudovirus Infection by an RBD-Independent Mechanism*. *Angew Chem Int Ed Engl* **2021**, *60* (18), 10279-10285. DOI: 10.1002/anie.202100316
(4) Percze, K.; Szakacs, Z.; Scholz, E.; Andras, J.; Szeitner, Z.; Kieboom, C. H.; Ferwerda, G.; Jonge, M. I.; Gyurcsanyi, R. E.; Meszaros, T. Aptamers for respiratory syncytial virus detection. *Sci Rep* **2017**, *7*, 42794. DOI: 10.1038/srep42794
